# Genome-wide identification, expression analysis and evolutionary relationships of the IQ67-domain gene family in common wheat (*Triticum aestivum* L.) and its progenitors

**DOI:** 10.1186/s12864-022-08520-w

**Published:** 2022-04-05

**Authors:** Qinglin Ke, Huifan Sun, Minqiang Tang, Ruihan Luo, Yan Zeng, Mengxing Wang, Yihan Li, Zhimin Li, Licao Cui

**Affiliations:** 1 College of Bioscience and Engineering, Jiangxi Agricultural University, Jiangxi, 330045 China; 2 College of Forestry, Hainan University, Hainan, 570228 China; 3 College of Agronomy, Jiangxi Agricultural University, Jiangxi, 330045 China; 4 Key Laboratory for Crop Gene Resources and Germplasm Enhancement, National Key Facility for Crop Gene Resources and Genetic Improvement, Institute of Crop Sciences, MOA, Chinese Academy of Agricultural Sciences, Beijing, 100081 China

**Keywords:** Wheat, *IQD* gene family, Expression profiles, Polyploidization, Asymmetric evolution

## Abstract

**Background:**

The plant-specific IQ67-domain (*IQD*) gene family plays an important role in plant development and stress responses. However, little is known about the *IQD* family in common wheat (*Triticum aestivum* L), an agriculturally important crop that provides more than 20% of the calories and protein consumed in the modern human diet.

**Results:**

We identified 125 *IQDs* in the wheat genome and divided them into four subgroups by phylogenetic analysis. The *IQDs* belonging to the same subgroup had similar exon–intron structure and conserved motif composition. Polyploidization contributed significantly to the expansion of *IQD* genes in wheat. Characterization of the expression profile of these genes revealed that a few *T. aestivum (Ta)IQDs* showed high tissue-specificity. The stress-induced expression pattern also revealed a potential role of *TaIQDs* in environmental adaptation, as *TaIQD-2A-2*, *TaIQD-3A-9* and *TaIQD-1A-7* were significantly induced by cold, drought and heat stresses, and could be candidates for future functional characterization. In addition, *IQD* genes in the A, B and D subgenomes displayed an asymmetric evolutionary pattern, as evidenced by their different gain or loss of member genes, expression levels and nucleotide diversity.

**Conclusions:**

This study elucidated the potential biological functions and evolutionary relationships of the *IQD* gene family in wheat and revealed the divergent fates of *IQD* genes during polyploidization.

**Supplementary Information:**

The online version contains supplementary material available at 10.1186/s12864-022-08520-w.

## Background

As an intracellular second messenger, calcium (Ca^2+^) is involved in plant growth and development as well as the regulation of abiotic and biotic stress responses [1]. The Ca^2+^ ion levels, induced by dose-dependent intracellular signals transduced through Ca^2+^ sensors, differ in their spatiotemporal properties associated with the strength and duration of environmental challenges [2]. There are four major categories of Ca^2+^ sensor proteins in plants, namely calmodulins (CaMs), CaM-like proteins (CMLs), calcineurin B-like proteins (CBLs), and Ca^2+^-dependent protein kinases (CDPKs) [3–5]. Upon sensing Ca^2+^, CaMs, CMLs and CBLs undergo conformational changes in their structures and interact with their target proteins to induce Ca^2+^ signals, while CDPKs contain an inherent kinase domain that can directly transduce the signal to the target protein when sensing Ca^2+^ signal [6]. CaMs are among the most common Ca^2+^ sensor proteins. Although lacking the catalytic activity, CaMs can interact and activate a wide spectrum of target proteins, and thereby play a crucial role in mediating physiological functions through their downstream target proteins. These target proteins include chaperones, metabolic enzymes, transcription factors, and kinases referred to as calmodulin-binding proteins (CaMBPs) [7]. CaMBPs are characterized by their calmodulin-binding domain (CaMBD), which consists of three conserved motifs, specifically, one Ca^2+^-independent motif (IQ motif), and two Ca^2+^-dependent motifs (l-5–10 motif and l-8–14 motif) [8]. The IQD proteins are common representatives of CaMBPs, characterized by a central region of 67 conserved amino acid residues, commonly known as IQ67 domain (IQD) protein families [9, 10]. The IQ67 domain consists of 1–3 copies of the IQ motif (IQxxxRGxxxR or [ILV]QxxxRxxxx[R, K]), 1–4 copies of the 1–5-10 motif ([FILVW]xxx[FILV]xxxx[FILVW]) and 1–4 copies of the 1–8-14 motif ([FILVW]xxxxxx[FAILVW]xxxxx[FILVW]) [11]. These features allow the IQ67 domain to form a basic amphiphilic helix structure, further endowing these proteins with specific roles [11].

In recent years, the research on the *IQD* gene family has attracted considerable attention in various model and non-model plants, such as *Arabidopsis*, *Oryza sativa* [11], *Solanum lycopersicum* [12], *Brachypodium distachyon* [13], *Glycine max* [14], *Populus trichocarpa* [15], *Zea mays* [16], *Cucumis sativus* [17], and *Brassica rapa ssp. pekinensis* [18]. Numerous studies have shown that the *IQD* genes are widely involved in microtubule-related signaling pathways, and play essential roles in various plant growth and development processes [19]. The microtubule-associated protein AtIQD5 controls cortical microtubule dynamics that promotes proper microtubule organization, and subsequent cell growth, cell shape formation and pavement cell morphogenesis [20, 21]. More recently, *Arabidopsis thaliana IQDs* were found to function as cellular scaffolds that facilitate preprophase band formation and cortical division zone establishment during symmetric cell division [22]. In rice, *OsIQD14* affects the grain shape by modulating microtubule cytoskeleton dynamics [23]. *IQD12*/*SUN* regulates tomato shape by redistribution of fruit mass, and also plays important roles in the growth of floral organ and leaf morphology [24, 25]. *SUN24* positively regulates seed germination by repressing the expression of two key ABA signaling genes (*SlABI3* and *SlABI5*) of the ABA signaling pathway in tomato [26]. *PdIQD10* regulates the biosynthesis of the second cell wall and impacts biomass formation in *Populus* [27]. Several IQD proteins are also implicated in the response to biotic and abiotic stresses in plants. *AtIQD1* can promote the accumulation of glucosinolates to reduce insect herbivory in *Arabidopsis* [10, 28]. In *Gossypium hirsutum*, knockdown of *GhIQD31* and *GhIQD32* negatively affected the responses of upland cotton to drought, salt, and cold stresses [29]. Overexpression of *BrIQD5* conferred drought stress tolerance to Chinese cabbage, possibly by interacting with CaMs and other drought‑related proteins [18].

As one of the most successful crops since the dawn of agriculture, common wheat (*Triticum aestivum* L.) has expanded its original habitat from a limited area within the Fertile Crescent to a wide range of environments worldwide, making it the most widely grown and consumed crop [30]. Common wheat (2n = 6x = 42, AABBDD) is an allohexaploid species derived from two rounds of hybridization between three distinct diploid species, and is an informative system for analyzing the asymmetric evolutionary patterns between different subgenomes [31]. It originated from two natural interspecific hybridization events within the genera *Triticum* and *Aegilops*, which had similar but distinct genome structure and gene content that diverged between 2.5 and 6 million years ago [32]. First, *Triticum urartu* (2n = 2x = 14, AA) hybridized with an uncertain grass that was highly similar to *Aegilops speltoides* (2n = 2x = 14, SS) to generate the tetraploid species of wild emmer or *Triticum turgidum* (2n = 4x = 28, AABB) at about 0.5 to 3 million years ago. The subsequent polyploidization combined the genomes of *Triticum turgidum* and *Aegilops tauschii* ((2n = 2x = 14, DD) to form the allohexaploid genome of *Triticum aestivum* at around 8000 years ago [33].

The completion of the genome sequence of hexaploid wheat has provided an opportunity to investigate gene families at the genome-wide level [34]. In this study, 125 *TaIQD* genes were identified from the wheat genome. Their phylogenetic relationships, conserved motif composition, intron–exon structure and physicochemical characteristics of their proteins were comprehensively analyzed. In addition, we evaluated the expression pattern of *TaIQDs* during the stage of post anthesis and embryo development, and in response to various stresses, in which the proteins encoded by some *TaIQD* genes might potentially play a crucial role in stress resistance. The evolutionary relationships with its progenitors were systematically assessed. Comparative genomic analysis of *TaIQD* genes in wheat and its progenitors revealed asymmetric evolution and expansion during wheat polyploidization, as evidenced by their biased gene gain and loss, homoeologous gene expression and nucleotide diversity. This study can serve as a useful reference for unravelling the evolution of *TaIQD* genes and will further contribute to functional gene cloning in wheat.

## Results

### Identification of *IQD* genes in wheat

This study identified a total of 125 *IQD* genes in the wheat genome (Table S1). Since there is no standard nomenclature for *IQD* genes in wheat, the wheat *IQD* genes were designated as *TaIQD-1A/1B/1D-1* to *TaIQD-7A/7B/7D-3* for the A, B and D subgenomes according to their chromosomal location and homoeologous relationships, and *TaIQD-U-1* to *TaIQD-U-2* for unanchored genes. As shown in Table S2, the length of the TaIQD proteins ranged from 339 (TaIQD-1B-6) to 2,388 (TaIQD-3B-5) amino acids (aa) with an average of 785.46 aa, with corresponding molecular weight from 37.1 to 271.15 kDa, and isoelectric point from 4.93 (TaIQD-2D-8) to 11.47 (TaIQD-4D-4). Noteworthy, all the IQD proteins has negative GRAVY values, indicating that these proteins have hydropathicity. The results of the subcellular localization revealed that 117 of the 125 (93.6%) TaIQDs were only found in the nucleus, the remaining TaIQDs were found in chloroplast, mitochondrion, endoplasmic reticulum, cell membrane and cell wall.

### Sequence alignment, phylogenetic analysis and structure of *TaIQDs*

**Fig. 1:**
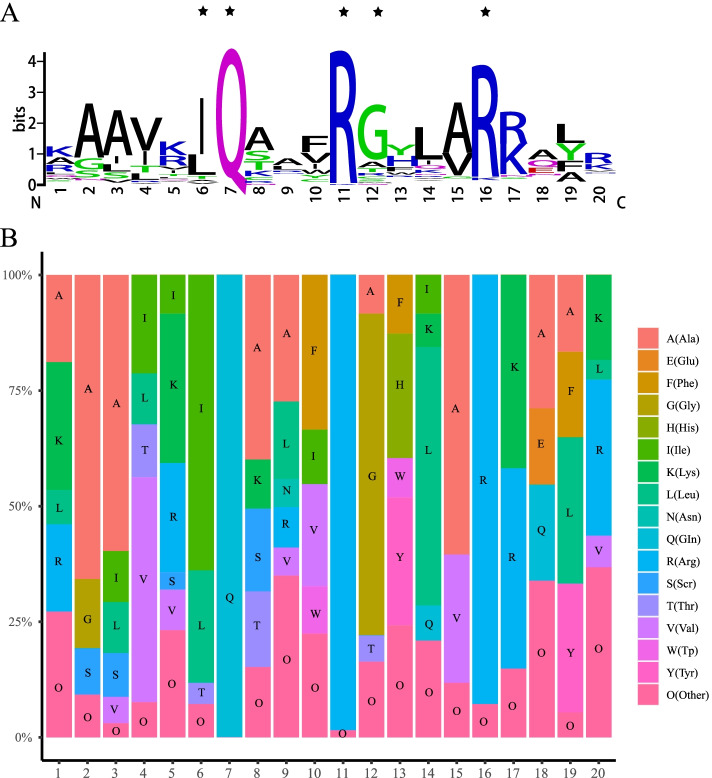
Conserved residue analysis of IQ domains. The height of each residue indicates the conservation rate. **A** Sequence logo generated by WebLogo, **B** Amino acid composition

The analysis of the domain conservation in the TaIQDs identified a total of 263 IQ motifs in wheat, with an average of 2.1 IQ motifs per protein, which was higher than that in rice (1.71), maize (1.65) and *Arabidopsis* (1.57). The length of the consecutive amino acid sequence of the IQ motifs ranged from 17 to 20 aa. As shown in Fig. 1, amino acid residues Ile-6, Gln-7, Arg-11, Gly-12, and Arg-16 were determined to be conserved amino acids with the conservative ratio of more than 60%. Notably, the conserved sequence ratio of Gln-7 was 100%, suggesting that this amino acid may be essential for the biological function of IQD proteins. Moreover, a similar pattern was observed in *Arabidopsis*, rice and maize (Table S3) [11, 16]. Besides IQ motifs, the search for calmodulin-binding sites revealed that TaIQDs have one to five CaM-binding sites with the consecutive amino acid length ≥ 7. Among them, TaIQD-2B-3 and TaIQD-2D-3 contain five CaM-binding sites, ranking as the most abundant CaM-binding domain containing TaIQDs. The predicted calmodulin interaction sites in 58 TaIQDs overlapped with the IQ motif (Table S4).

**Fig. 2:**
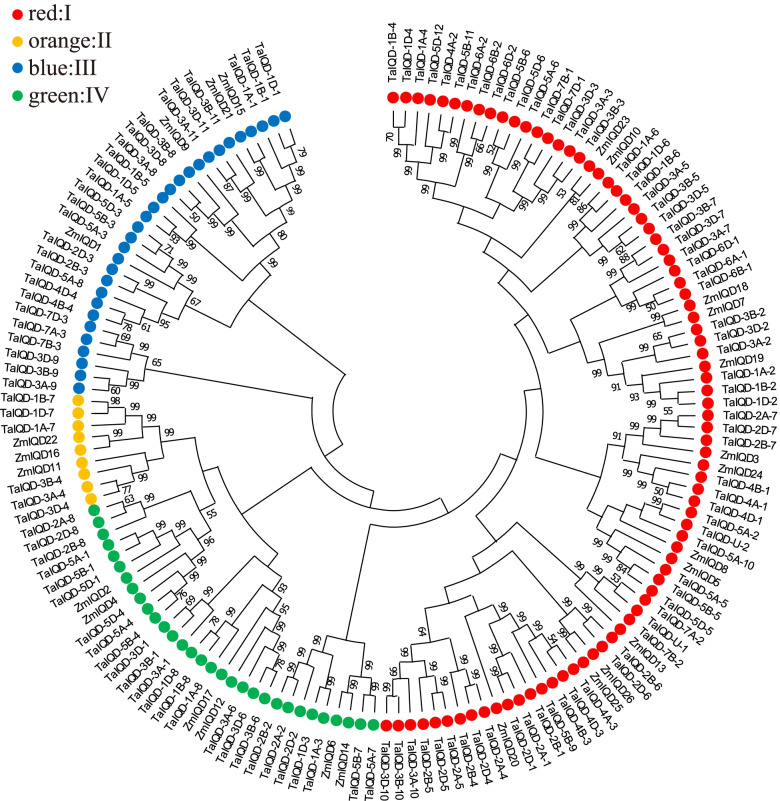
Phylogenetic tree analysis of IQD proteins from wheat and maize. The four IQD subgroups were marked with different colors. Red for subgroup I, orange for subgroup II, blue for subgroup III, green for subgroup IV

In order to assess the evolutionary relationships of wheat *TaIQD* genes, an unrooted phylogenetic tree was constructed based on the alignment of the full-length sequence of IQD proteins from wheat (125 proteins) and maize (26 proteins). The TaIQDs were classified into four subgroups designated as I, II, III and IV on the basis of the classification principle used in maize (Fig. 2). The ratio of memberships within each subgroup in wheat was similar to that in maize, *Arabidopsis* and rice [11, 16]. Specifically, subgroup I had the most IQD proteins (68), followed by subgroup III (26) and subgroup IV (25), while subgroup II had the fewest with only six members (*TaIQD-3A/3B/3D-4* and *TaIQD-1A/1B/1D-7*) (Table S5).

**Fig. 3:**
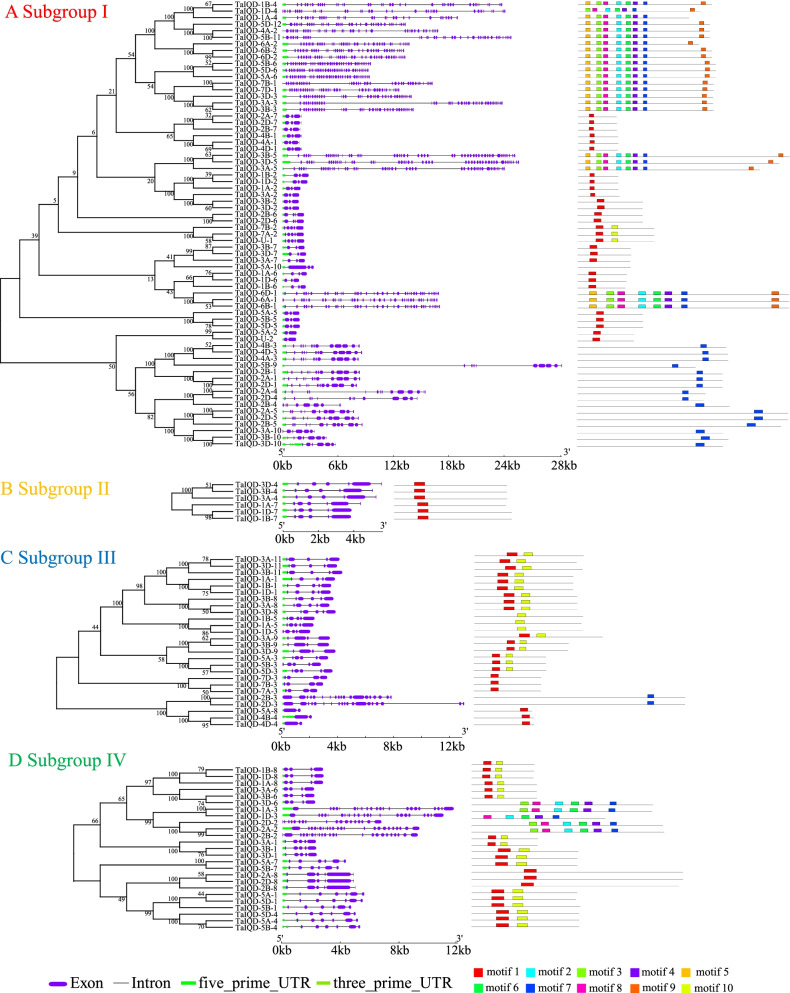
Phylogenetic relationships, exon–intron organization, and motif composition of *TaIQDs*. Exons and introns are indicted by grep rectangles and black lines. Conserved motifs are represented by colored boxed. **A** Subgroup I, **B** Subgroup II, **C** Subgroup III, **D** Subgroup IV

The exon–intron structure could also have certain reference value to understand the relative relationships of *TaIQD* genes. As shown in Fig. 3, the number of exons of *IQD* genes ranged from 2 (*TaIQD-5A-2*) to 53 (*TaIQD-3B-5*). The average exon length was 184.6 bp, whereas the intron length varied from 63 to 19,468 bp with an average length of 356.18 bp, indicating that the noncoding regions were subjected to lower selection pressure, thereby exhibited higher sequence diversity. It is noteworthy that *TaIQDs* grouped within the same subgroup shared a similar exon–intron structure and number of exons.

Further analysis of the motif composition of TaIQD proteins predicted a total of 10 conserved motifs (Figure S1). The most abundant motif 1 and motif 7 were exclusively present in 75 and 46 TaIQDs, respectively. Both motifs represented the core sequence region of the IQ motifs. Motif 1 represented the conventional IQ motif (IQxxxRGxxxR), whereas motif 7 was relaxed version of the IQ motif ([IL]QxxxRxxxxR). Motifs 2, 3, 4, 6 and 8 were uniquely present in Group I and Group IV. Subgroup II only contained motif 1, possibly due to its conserved exon–intron structure. In addition, a conserved motif arrangement was observed within each subgroup, but different subgroups contained their specific organization, and we thus inferred that *TaIQDs* have conserved and diverse functions.

### Syntenic relationships of *TaIQD* genes in wheat and its relatives

The *TaIQD* genes were found to be unevenly distributed along the wheat chromosomes. Out of the 125 *IQD* genes identified in the wheat genome, a total of 123 *TaIQDs*, comprising 41 for A, 42 for B and 40 for D subgenomes, were mapped to the chromosomes (Figure S2). Most of the *TaIQDs* (81.6%) had three copies associated with subgenomes A, B and D. Group 3 chromosomes contained more *IQD* genes than other chromosomes, with 11 *IQD* genes in the A, B and D subgenomes, respectively. In addition, group 6 chromosomes had the lowest number of *IQD* genes, with only two members for each subgenome. Most of the *TaIQDs* were located at the distal regions of the chromosome. Genetic and cytological studies have demonstrated that recombination events predominately occur at distal regions of the chromosome, but suppressed at pericentromeric regions [35, 36].

It should be noted that *TaIQD-U-2* and *TaIQD-U-1* were not located on definite chromosomes. Given that *TaIQD-U-1* showed homology with *TaIQD-7A-2* and *TaIQD-7B-2*, and *TaIQD-U-2* was homologous with *TaIQD-5A-2*, we thus speculated that *TaIQD-U-1* and *TaIQD-U-2* were located at the middle of chromosome 7D and the top of chromosome 5D, respectively.

As the representative allopolyploid species, the genomic duplication of A, B and D subgenomes play an indispensable role in the expansion of the total gene dose within the genome. For this reason, we further performed the analysis of the syntenic relationships among different subgenomes. Ultimately, a total of 87 gene pairs consisting of 101 *IQD* genes were found to be syntenic genes. There were 25 homoeologous gene groups with the three complete copies associated with A, B and D (Figure S3). The *TaIQD-5A/5B-7*, *TaIQD-1B/1D-7*and *TaIQD-1A/1D-3* syntenic groups were only observed for that between A and B, B and D, and A and D, respectively. However, no tandem duplication was detected in our study, suggesting that genomic polyploidization led to the expansion of the *IQD* family in wheat. The Ka/Ks ratios for the 87 *TaIQD* syntenic gene pairs were estimated and the values varied from 0.0234 to 0.5865, with an average value of 0.1677, suggesting that the *IQD* gene family experienced strong purifying selection pressure (Table S6).

**Fig. 4:**
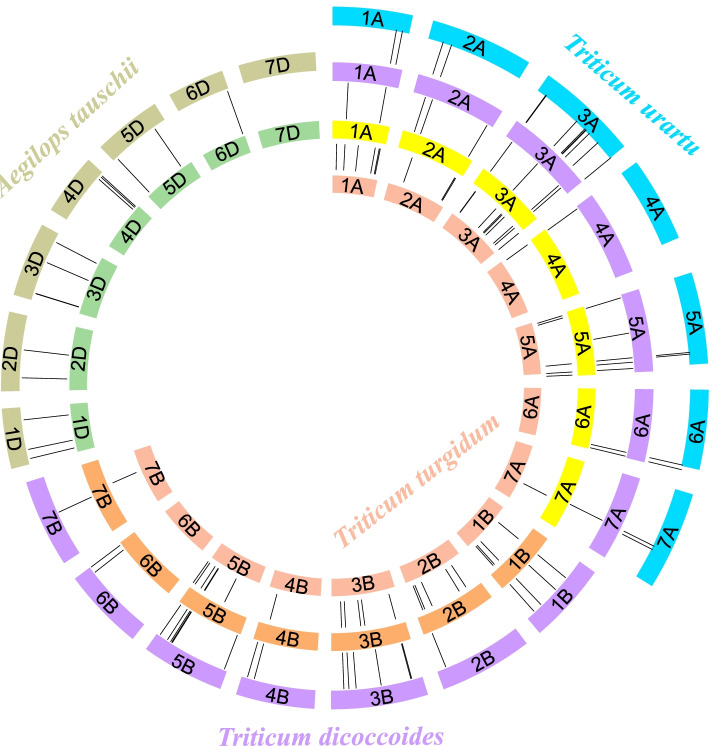
Syntenic analysis of *IQD* genes among common wheat and its progenitors. The genomes of *Triticum urartu*, *Triticum dicoccoides*, *Triticum trugidum* and *Aegilops tauschii* surround the central *Triticum aestivum*. Syntenic gene pairs are linked by lines

To further elucidate the evolutionary mechanism of *IQD* genes in wheat and its progenitors, a unified identification standard as described for wheat was used to identify the *IQD* genes in other species. A total of 232 *IQD* genes were identified, comprising 36 from *Triticum urartu*, 76 from *Triticum dicoccoides*, 78 from *Triticum turgidum* and 42 from *Aegilops tauschii* (Fig. 4, Table S7). For the A subgenome, 36 *IQD* genes from *Triticum turgidum* showed syntenic relationships with those of wheat, followed by *Triticum dicoccoides* (32), and *Triticum urartu* (28). It was found that 20 *TaIQD*s of the A subgenome were also present in the three related species. We thus speculate that since these genes may have important biological functions, they have a definite conservation rate during evolution. In addition, seven *IQD* genes (*TaIQD-1A-1*, *TaIQD-1A-2*, *TaIQD-2A-8*, *TaIQD-3A-5*, *TaIQD-3A-9*, *TaIQD-3A-10*, *TaIQD-5A-1*) were identified as homologs between *Triticum aestivum* and *Triticum dicoccoides* as well as between *Triticum aestivum* and *Triticum turgidum*. However, no homologous genes were found between *Triticum aestivum* and *Triticum urartu*, suggesting that these homologous pairs might be formed after wheat tetraploidization. For the B subgenome, 36 *IQD*s were identified as syntenic gene pairs between *Triticum aestivum* and *Triticum dicoccoides*, and 37 between *Triticum aestivum* and *Triticum turgidum*. The *TaIQD-3B-2* formed no homologous gene pairs or showed homologous relationships with other genes in other species. For the D subgenome, 40 *TaIQDs* showed homologous relationships with 39 *IQD* genes in *Aegilops tauschii*. Noteworthy, one *IQD* in *Triticum aestivum* and three *IQDs* in *Aegilops tauschii* showed no collinearity with the other species, suggesting that these genes might experience gene acquisition, gene loss or chromosome translocation after wheat polyploidization.

Furthermore, the calculation of the Ka/Ks ratios revealed the orthologous relationships of *IQD* genes between wheat and its relatives (Table S8). The Ka/Ks ratios of 2, 10, 14 and 15 homologous gene pairs values were found to be higher than 1 in *Triticum urartu*, *Aegilops tauschii*, *Triticum dicoccoides*, *Triticum turgidum*, suggesting that these genes might undergo positive selection during the evolutionary process. In contrast, the rest of the homologous gene pairs had negative Ka/Ks ratios, suggesting that most of the *IQD* genes were subjected to purifying selection pressure.

### Expression profiling of *TaIQD* genes in various stages

**Fig. 5:**
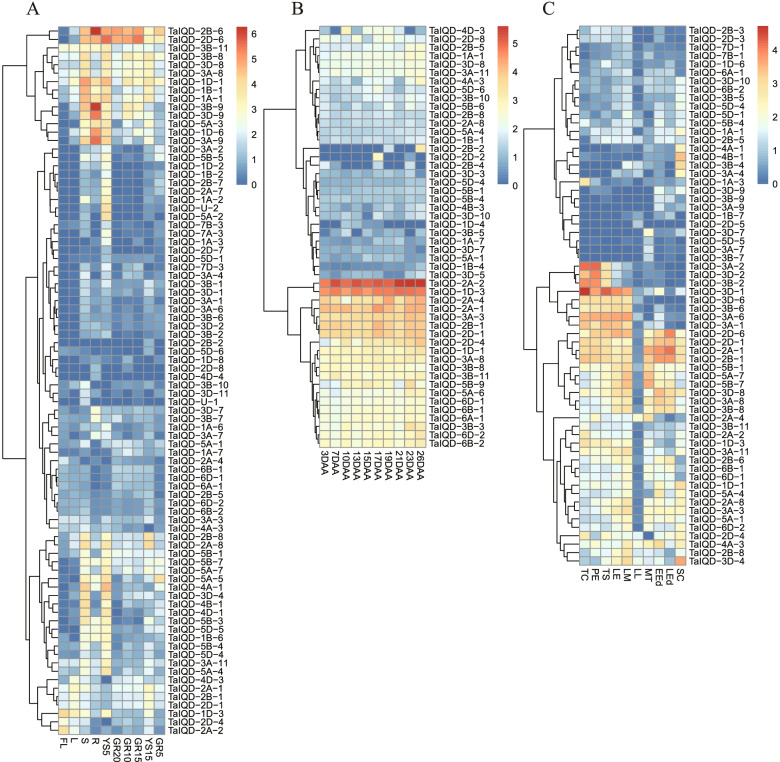
Expression pattern of *TaIQDs* during different developmental stage. Red indicates high expression and blue indicates low expression. The left represents gene clusters. **A** different developmental stages. FL: flag leaf at heading stage, YS5: young spike at early booting stage, YS15: spike at heading stage. R, S, and L represent root, stem, and leaf of five-leaf stage seedling, GR5, GR10, GR15, and GR20 represent grain at 5, 10, 15, and 20 days post-anthesis, respectively. **B** 3 to 26 days after anthesis, **C** ten different stages during embryo development. TC: two cells, PE: pre-embryo, TS: transition, LE: leaf early, LM: leaf middle, LL: leaf late, MT: mature, EEd: early endosperm, Led: late endosperm, SC: Pericarp

The investigation of the potential biological functions of *TaIQDs* through the analysis of the expression profiles of *TaIQDs* using publicly available RNA-seq data identified a greatly divergent expression pattern in different developmental stages or tissues. *TaIQD-4D-4* and *TaIQD-2D-8* were preferentially expressed in root (Fig. 5A). High expression levels of *TaIQD-5A-2, TaIQD-7A-3*, *TaIQD-2B-7*, and *TaIQD-1D-2* were found in stem and five-days young spike. Moreover, the expression patterns of *TaIQDs* at ten time points after anthesis were also identified (Fig. 5B). The expressed genes were divided into three major groups. The *TaIQDs* in the first group, such as *TaIQD-2A-2*, *TaIQD-1D-3*, *TaIQD-2A-1*, *TaIQD-2A-4*, *TaIQD-2B-1*, *TaIQD-2D-1*, *TaIQD-3A-3*, showed relatively high expression level at most time points, suggesting that these genes may play critical roles during the whole anthesis period in wheat. In addition, we also found several *TaIQD*s with high tissue-specificity. For example, *TaIQD-2D-2* exhibited preferential expression at 17 DAA, while *TaIQD-2B-2* was unevenly expressed at 26 DAA time point.

Further analysis of the expression profiles at ten different time points during embryonal development (Fig. 5C) showed that 64 *TaIQD* genes were expressed in at least one time point. The highly tissue-specific *IQD*s were also identified. For instance, *TaIQD-3A/3B/3D-2* showed expression bias in two cell types, pre-embryo, and transition stages, implying that the three homoeologous genes may participate in early embryogenesis. In contrast, *TaIQD-2A/2B/2D-1* showed relatively high expression in the late endosperm stage. *TaIQD-3A/3B-4*, *TaIQD-4A/4B-1* and *TaIQD-3D-4* were mainly expressed in seed coat.

### Expression profiling of *TaIQD* genes in response to various stresses

**Fig. 6:**
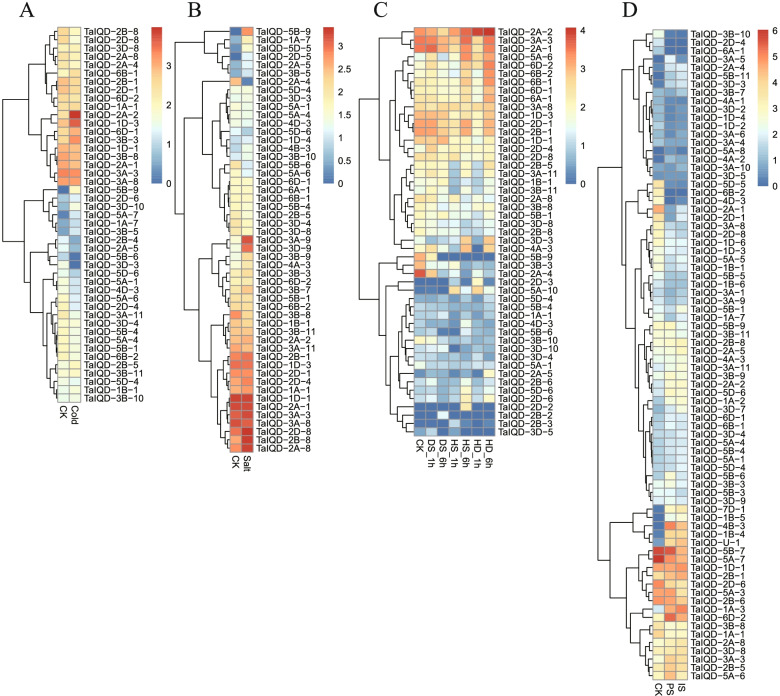
Expression analysis of *TaIQDs* in response to various abiotic stress. Red indicates high expression and blue indicates low expression. The left represents gene clusters. **A** Cold, **B** Salt, **C** Heat with drought, **D** Phosphorus and iron starvation. DS: drought stress, HS: heat stress, HD: heat with drought stress, PS: Phosphorus starvation, IS: Iron starvation

We also investigated the biological function of *TaIQD* genes in the response to various abiotic stresses, specifically cold, salt, drought/heat, and metal starvation. The results showed that 45 genes expressed in response to cold stress (Fig. 6A). The *TaIQD-3D-10*, *TaIQD-3B-5*, *TaIQD-5A-7*, and *TaIQD-5B-9* genes were markedly upregulated. Remarkably, *TaIQD-5A-7* showed about 6.62-fold higher expression level compared to the control. *TaIQD-5B-9* was not expressed under untreated condition, but was markedly expressed in response to cold stress. Additionally, *TaIQD-3D-3*, *TaIQD-5B-6*, *TaIQD-2B-4* and *TaIQD-2A-5* were weakly expressed when subjected to cold treatment. Under salt stress (Fig. 6B), seven genes showed upregulated expression patterns. *TaIQD-5D-5* and *TaIQD-1A-7* showed 6.47 and 3.42-fold upregulation after exposure to salt. Moreover, the expression of *TaIQD-2D-5* and *TaIQD-5B-9* were induced in response to salt stress. When the plants were subjected to the combined stresses of drought and heat (Fig. 6C) with the following six treatment and time point conditions (DS_1h, DS_6h, HS_1h, HS_6h, HD_1h and HD_6h), a total of 2, 2, 3, 7, 2 and 5 *TaIQD* genes were upregulated and 2, 7, 13, 8, 10, and 7 *TaIQD* genes were downregulated, respectively. *TaIQD-5A-6* showed 2.13 and 2.02-fold upregulation under the HS_6h and HD_6h treatments, and *TaIQD-2A-5* showed more than twofold upregulation under the DS_1h, DS_6h, HS_6h and HD_6h treatments. The expression profiles of *TaIQDs* under phosphorus and iron deprivation were also determined (Fig. 6D). Remarkably, the expression levels of *TaIQD-4A-2*, *TaIQD-1B-4*, *TaIQD-5B-11*, *TaIQD-3D-3*, *TaIQD-7D-1*, and *TaIQD-U-1* showed more than fivefold upregulation than those of their respective control. The rest of the *TaIQDs* showed weak or moderate expression levels, suggesting that only a few genes are involved in the response to various stresses in wheat.

In order to gain a deep understanding into the expression of *TaIQD* family genes in response to multiple stresses, 9 *TaIQD* genes from four different subgroups were randomly selected to study their expression profiles under salt, drought, cold and heat stresses by qRT-PCR analysis (Figure S4). Under salt stress, the selected *TaIQDs* were upregulated at different time points. For example, *TaIQD-2A-2* and *TaIQD-3A-9* were upregulated at all time points and reached their maximum expression level at 6 h and 1 h, respectively. The expression of *TaIQD-1A-7* peaked at 12 h with a 6.96-fold upregulation. At different time points of the cold stress treatment, two *TaIQDs* (*TaIQD-2A-2* and *TaIQD-5B-9*) were upregulated, whereas *TaIQD-3B-5* was downregulated at all time points. Meanwhile, some *TaIQD* genes showed variable expression profiles at different time points. For instance, *TaIQD-5A-6* and *TaIQD-3D-9* were downregulated at 6 h, but upregulated at the remaining time points. In addition, the expression levels of the selected *TaIQDs* were analyzed after drought stress treatment. The expression levels of *TaIQD-3B-5*, *TaIQD-3D-10* and *TaIQD-3D-9* were suppressed compared with those of the control. The expression of levels of *TaIQD-1A-7* and *TaIQD-3A-9* were significantly upregulated, and peaked at different times. Specifically, the expression of *TaIQD-1A-7* peaked at 1 h and was upregulated 4.84-fold, whereas the expression of *TaIQD-3A-9* was initially slightly upregulated and peaked at 12 h. The results of the qRT-PCR analysis revealed that heat treatment had a marked effect on the expression patterns of *TaIQDs*. With the exception of *TaIQD-3B-5* and *TaIQD-5B-9*, whose expression was inhibited compared with the control, the expression levels of a total of six *TaIQDs* (*TaIQD-1A-7*, *2A-2*, *3A-9*, *3B-11*, *3D-9* and *5A-6*) peaked at 24 h, suggesting that these *TaIQDs* might primarily function in the terminal stage in the response to heat injury. Notably, the expression of *TaIQD-2A-2*, *TaIQD-3A-9* and *TaIQD-1A-7* was significantly altered in response to salt, cold, heat and drought stresses, indicating that they might be excellent targets for the molecular breeding of wheat.

### Cis-regulatory elements and co-expression network analysis of TaIQDs

As the region containing the transcription factor binding site that initiates transcription, the promoter plays an essential role in controlling the expression of genes that are involved in plant organogenesis, hormone signal transduction and stress responses. In total, six hormone-related *cis*-regulatory elements associated with gibberellin (GA), auxin, methyl jasmonate (MeJA), ethylene, salicylic acid (SA) and abscisic acid (ABA) were detected (Table S9 and Figure S5). Except for *TaIQD-2B/2D-3*, the majority of the *TaIQDs* had more than 13 hormone- or stress-responsive related *cis*-elements. In particular, gibberellin- (GARE-motif, P-box, TATC-box), MeJA- (CGTCA-motif, TGACG-motif), ABA- (ABRE), auxin- (TGA-element, AuxRR-core), ethylene- (ERE) and salicylic acid- (SARE, TCA-element) were found in 59, 104, 105, 58, 19, and 32 *TaIQD*s, respectively. Abundant hormone-responsive *cis*-regulatory elements were enriched in the promoter regions of *TaIQD-1B-4*, *TaIQD-5A-4*, *TaIQD-2B-4*, and *TaIQD-3B-6*. In addition, numerous abiotic stress *cis*-elements were also found, such as low-temperature responsive element LTR (53 genes), drought responsive element MBS (50 genes), salinity, as well as dehydration responsive elements DRE (three genes), DRE core (64 genes) and DRE1 (17 genes). Additionally, three kinds of biotic stress related *cis*-regulatory elements were also detected, including defense responsive TC-rich repeat elements (27 genes), wounding responsive element WUN-motif (23 genes) and wounding responsive element 3 (WRE3) (82 genes). These results implied that *TaIQD* genes might play critical roles in biotic and abiotic stresses, and might be involved in hormone stimulus responses.

MicroRNAs (miRNAs) can direct the cleavage of target mRNA or translation inhibition to regulate plant development and response to environmental fluctuations [37]. In this study, the putative miRNAs targeting the mRNAs of *TaIQDs* were predicted by psRNATarget. A total of 20 miRNA-*TaIQD* putative targeting relationships comprising 13 miRNAs and 13 *TaIQDs* were predicted with more than 90% sequence alignment (Table S10). Specifically, tae-miR9653a precisely binds to *TaIQD-1A-7* with 100% alignment. All the miRNAs silenced the post-transcriptional expression of *TaIQDs* through transcript cleavage. Moreover, except for miR1120c-*TaIQD-2D-5*, the rest of the miRNA-*TaIQD* interactions were found to act upstream of the IQ domains. Overall, these results suggest that miRNAs may have crucial roles in the post-transcriptional regulation of the expression of *TaIQD*, and further research on the miRNA-mediated interaction relationships will provide valuable information to understand the functional roles of *TaIQDs* in plant growth and development as well as stress responses.

**Fig. 7:**
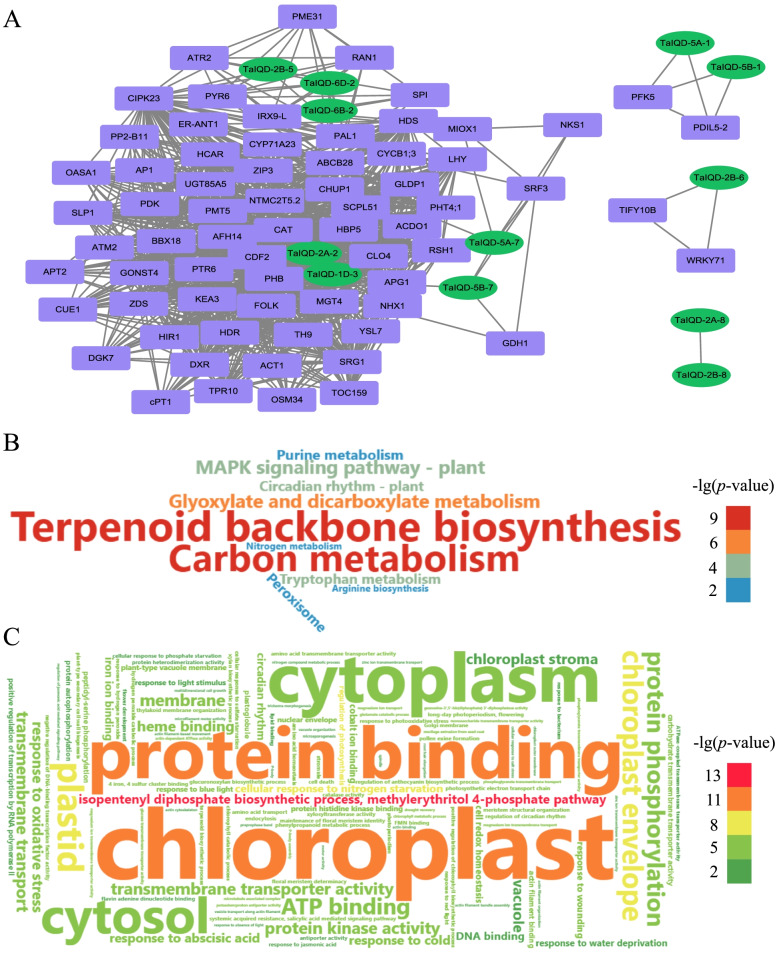
The co-expression network between *TaIQDs* and other wheat genes based on WGCNA analysis. **A** WGCNA analysis, **B** KEGG enrichment of *TaIQD* co-expressed genes, **C** GO enrichment of *TaIQD* co-expressed genes

To investigate the regulatory functions of *TaIQDs* associated with other wheat genes, the available 110 RNA-seq samples were used to construct a co-expression network (Fig. 7A). The network consisted of a total of 913 links consisting of 12 *TaIQDs* and 68 other genes. Among them, the highly connected *TaIQD-1D-3* and *TaIQD-2A-2*, located at the core node position, were co-expressed with 53 (66.25%) and 13 (16.25%) related genes, respectively, suggesting that these two *TaIQDs* might play a central role in the regulatory network. Two *TaIQD* genes (*TaIQD-2B-5* and *TaIQD-6D-2*) had co-expression relationships with *PYR6, CIPK23*, *PME31*, *PAL1*, and *ATR2*. The genes co-expressed with *TaIQDs* were significantly enriched in functional categories that included terpenoid backbone biosynthesis, biosynthesis of secondary metabolites, metabolic pathways, carbon metabolism and other KEGG pathways (Fig. 7B). GO enrichment analysis of the *TaIQD* co-expressed genes revealed that they were most enriched in the terms related to multiple developmental process (Fig. 7C), such as cellular response to nitrogen starvation (GO:0,006,995), regulation of photosynthesis (GO:0,010,109), response to cold (GO:0,009,409), response to abscisic acid (GO:0,009,737), and cellular response to salt stress (GO:0,071,472).

### Nucleotide variation and population structure analysis of *TaIQD* genes

The genetic landscapes with the genera *Triticum* and *Aegilops* have been comprehensively analyzed at the whole-genome level [30], but studies of the nucleotide variation patterns of *TaIQDs* are rather limited. By taking advantage of the cutting-edge analysis tools of whole-genome sequencing datasets, the nucleotide variation analysis uncovered 5,145 *TaIQD*-related SNPs, including 1,430, 1,297 and 2,418 for the A, B and D subgenomes, respectively. The majority of the SNPs were located within the upstream (38.46%) or downstream (32.69%) regions, followed by the intronic regions (17.47%), while only 10.65% SNPs were in exonic regions (Table S11). Within the coding regions, we observed 4.30% synonymous and 2.93% non-synonymous SNPs with a synonymous versus non-synonymous ratio of 1.46.

**Fig. 8:**
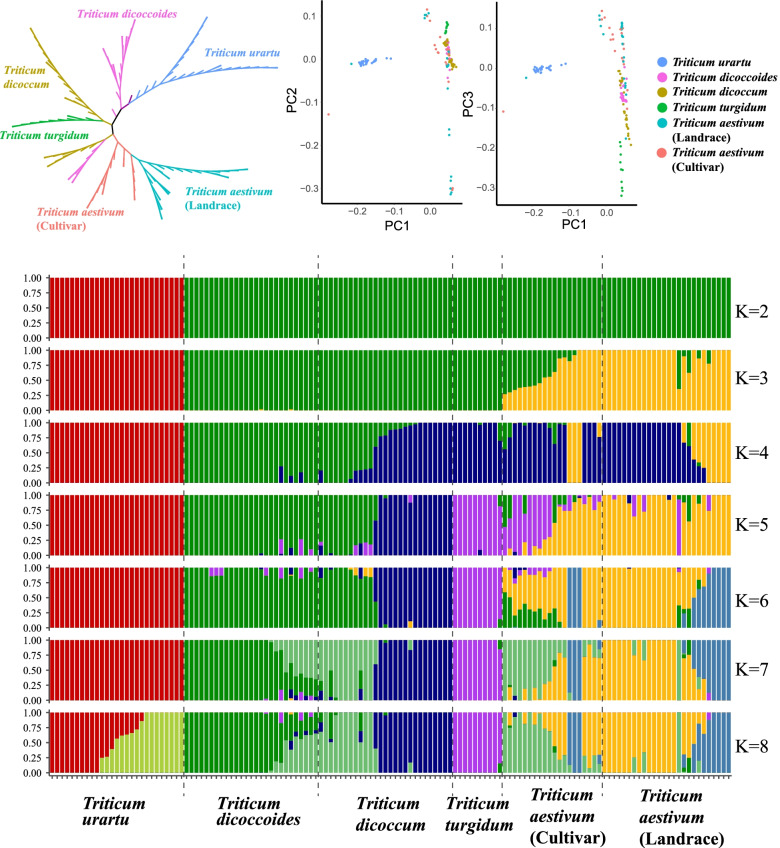
Phylogenetic relationships, PCA and population structure analysis for the group A genomes based on *TaIQD*-related SNPs. The SNPs from the A subgenome/genome of *Triticum urartu*, *Triticum dicoccoides, Triticum dicoccum*, *Triticum turgidum* and *Triticum aestivum* were used. **A** Neighbor-joining phylogenetic tree, **B** PCA analysis of PC1 *vs* PC2, **C** PCA analysis of PC1 *vs* PC3, **D** Population structure was estimated by ADMIXTURE with the K range from 2 to 8

The evolutionary relationships and population structure of the different subspecies were further studied at the sub-genomic level. For the A subgenome, principal component analysis (PCA) showed that the first principal component accounted for 61.4% of the total variance and mainly distinguished the *Triticum urartu* from the other species, whereas *Triticum aestivum* (landrace) was mainly distinguished by the second principal component (15.93% of total variance), and *Triticum turgidum* was distinguished by the third (Fig. 8B and C, Table S12). A more obvious subgroup that included from top to bottom *Triticum urartu*, *Triticum dicoccoides*, *Triticum dicoccum*, *Triticum turgidum* and *Triticum aestivum*, was identified through the phylogenetic tree (Fig. 8A). Admixture analysis provided similar evidence (Fig. 8D). When K = 2, the species *Triticum urartu* was firstly recognized. With the increase of K to 3, the landraces and cultivars of common wheat were separated from the others. With the continuous increase of the K value, a certain proportion of gene flow between common wheat and its progenitors was observed, indicating the continuous gene flow between its diploid and tetraploid ancestors and hexaploid wheat during and after the process of polyploidization. The nucleotide diversity increased gradually from the diploid wheat (*Triticum urartu*) to tetraploid wheat (*Triticum dicoccoides*, *Triticum dicoccum* and *Triticum turgidum*) and then to hexaploid wheat (*Triticum aestivum*). The genetic diversity of *Triticum dicoccum* and *Triticum turgidum* populations was basically the same, but a significant genetic loss (40.2% reduction) occurred in the *Triticum dicoccoides* population during domestication. The fixation index (*F*_*st*_) is an important index used to evaluate gene flow intensity and population differentiation [38]. If the *F*_*st*_ value is larger than 0.25, populations are considered to be extremely divergent [39]. In this study, the *F*_*st*_ values between *Triticum urartu* and other populations were larger than those within the other populations, which was consistent with the results of the phylogenetic relationships with the deviated cluster groups of the *Triticum urartu* population.

**Fig. 9:**
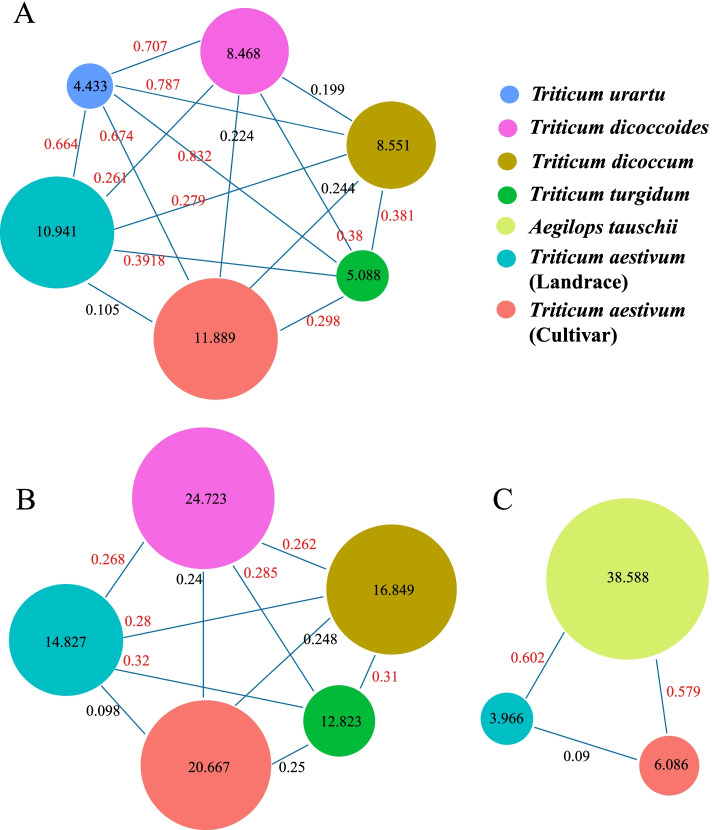
Distribution of nucleotide diversity (π × 10^2^) and *F*_*st*_ values across the group A, B and D genomes, respectively. The *F*_*st*_ values larger than 0.25 are marked with red. **A** A subgenome, **B** B subgenome, **C** D subgenome

For the B subgenome, all accessions were assigned to five subgroups according to their biological sources. The first, second and third components mainly captured the difference of *Triticum dicoccoides*, *Triticum dicoccum* and *Triticum turgidum*, respectively. Within the phylogenetic tree, the *Triticum dicoccum* population was definitely separated from the others, but there was no obvious boundary between the landrace and modern cultivar accessions for both the tetraploid wheat and hexaploid wheat. The same population affinities were recovered in the stacked bar based on the Admixture analysis. When K = 2, a genetic admixture was observed for *Triticum dicoccoides* and *Triticum turgidum*. However, it was not until K increased to 4 that *Triticum turgidum* formed a relatively independent subgroup. When K increased above 5, the landrace and hexaploid wheat gradually diverged, but there was still obvious genetic admixture between the two populations. We further evaluated the genetic diversity of the B subgenome for different populations. The nucleotide diversity of *TaIQDs* decreased continuously from *Triticum dicoccoides* (0.2472) to *Triticum dicoccum* (0.1685), and ultimately to *Triticum turgidum* wheat (0.1282) (Fig. 9, Figure S6).

We also profiled the nucleotide variation atlas of *TaIQDs* for the D subgenome. As described in subparagraph A of Figure S7, a significant genetic divergence was observed between the D subgenome of hexaploid wheat and its ancestral species *Aegilops tauschii*. Identical results were obtained when the high *F*_*st*_ values were calculated for wheat varieties *versus Aegilops tauschii* (0.602) and wheat landraces versus *Aegilops tauschii* (0.579), which suggested that these populations were highly differentiated between each other. Moreover, the average nucleotide diversity of *Aegilops tauschii* ranked the highest among the studied populations of different subgenomes. However, the nucleotide diversity of the D subgenome decreased ~ 85% from the ancestral species to hexaploid wheat. In summary, the evolutionary patterns of *TaIQD* genes provide novel insights into the process of wheat polyploidization, which might be useful in wheat genetic research and germplasm resource utilization in the future.

## Discussion

### The first characterization of *IQD* gene family in wheat

The members of the plant-specific *IQD* gene family, encoding a class of calmodulin-binding proteins involved in calcium signaling pathways, play essential roles in plants coordinating a wide range of developmental processes and responses to environmental stimuli [11]. Wheat is one of the most important cultivated grain crops worldwide, contributing approximately a fifth of the food consumption for the majority of the human populations [40]. In total, 125 non-redundant *IQD* genes were identified in the wheat genome. The number of *IQD* genes in wheat is more than three times higher than that in *Arabidopsis thaliana* (33) [11], *Brachypodium distachyon* (23) [13], *Brassica rapa ssp. pekinensis* (35) [18], maize (26) [16], *Oryza stative* (29) [11], *Populus trichocarpa* (40) [15], potato (23) [41], and tomato (34) [12]. Compared with its progenitors, the number of *IQD* in *Triticum aestivum* is approximately three times higher than in *Triticum urartu* (36) and *Ae. tauschii* (42), whereas the ratio of *TaIQDs* between *Triticum aestivum and Triticum dicoccoides* or between *Triticum aestivum* and *Triticum turgidum* was approximately 1.5. This finding is consistent with those of previous studies on various gene families, including the *TaPRX* (374 in *Triticum aestivum*, 159 in *Triticum urartu* and 169 in *Aegilops. tauschii*) [42], WOX (43 in *Triticum aestivum*, 23 in *Triticum dicoccoides*, 28 in *Triticum. turgidum*, 16 in *Triticum urartu* and 13 in *Aegilops tauschii*) [43] and *Hsp70* (113 in *Triticum aestivum*, 79 in *Triticum dicoccoides* and 30 in *Aegilops tauschii*) [44] gene families. This phenomenon can be explained by the two rounds of polyploidization that directly led to the expansion of the *IQD* genes in wheat.

A comparative genomic analysis of *IQD* gene family members from wheat and maize was performed to investigate the phylogenetic relationships. The phylogenetic analysis revealed that TaIQD proteins fell into four subgroups within the tree. Subgroup I was the largest subgroup, followed by subgroup III, which was in agreement with the results in maize, *Arabidopsis* and rice [11, 16]. The TaIQD proteins in the same subclade tend to cluster with each other more than proteins from the same species in different subclades. Meanwhile, genes within the same subgroup had a similar gene structure and conserved motif composition. Motif 1 and motif 7, which were the most common motifs, represented the conventional IQ67 motif (IQxxxRGxxxR) and a more relax version ([IL]QxxxRxxxxR). In addition, the subgroup specific motifs were also identified, which may be related to the functional diversification of *TaIQDs*.

### The potential function of *TaIQDs* might be in plant growth, development and response to various stresses

The biological functions of IQD proteins have been extensively studied in many plants, especially model plants [19]. The elucidation of the expression patterns of *TaIQDs* during plant growth and development will provide new insights into the potential functions of their proteins. It was recently demonstrated that *OsIQD14* acts as a key regulator in cortical microtubule rearrangements thereby affecting the grain shape [23]. *TaIQD-U-1* (*or TaIQD-7D-2*), which is homologous to *OsIQD14*, showed high tissue-specificity with a τ ≈ 1 and biased expression in the stem of five-leaf-stage seedlings. These results suggested that *TaIQD-U-1* might perform different functions in wheat. Moreover, there was a relatively high expression level of *TaIQD-1D-1* during the whole stage of anthesis. Its orthologous gene *AtIQD1* encodes a protein found in microtubules that interacts with the KLCR1 (kinesin light chain related 1) protein to expedite cellular transportation [10]. Our results suggest that *TaIQD-1D-1* might be involved in mediating the transition from vegetative to reproductive development in barley. In *Arabidopsis*, *AtIQD5* was reported to be involved in the process of cell shape morphogenesis, whereas its orthologous gene in wheat, *TaIQD-5B-1* was preferentially expressed in embryonal leaf middle and mature stages [21], implying that *TaIQD-5B-1* might be involved in these processes. Homologous analysis reveals the potential function of *TaIQD*s, but to ascertain the function of *TaIQD*s further extensive experimental work is needed.

As sensible organisms that are not able to move, terrestrial plants are often exposed to a wide array of adverse challenges, such as drought, high salinity, extreme temperatures, and pathogen infection [45]. To adapt to such environmental stimuli in an appropriate manner, plants have evolved complex signal transduction pathways that enable them to perceive stress signal and coordinate their growth and development [46]. In this study, we identified several cold, salt, drought/heat, and metal starvation induced *IQD* genes. Most *TaIQDs* tend to be induced by drought/heat rather than cold stress. For instance, *TaIQD-2B-2*, *TaIQD-2D-3*, and *TaIQD-5A-10* were significantly upregulated by more than 20-fold after exposure to drought/heat. In Chinese cabbage, over-expression of *BrIQD5* conferred plants drought tolerance, while *BrIQD5*-silenced plants exhibited drought sensitivity [18]. *TaIQD-3A-9*, one of the BLAST hits of *BrIQD5* in wheat, displayed significant up-regulated expression patterns at 1, 6, 12, and 24 h under drought stress. Abundant MBS *cis*-acting elements associated with drought inducibility within the promoter regions were also identified. These results suggested that *TaIQD-3A-9*, which showed homology to *BrIQD5*, might be involved in the response to drought stress. Although the identified candidate *IQD* genes could serve as targets for subsequent genetic isolation and functional investigation in wheat, further studies are needed to determine the biological functions of these *TaIQDs*.

As a systems biology approach for determining the potential interactions among genes, WGCNA is an effective method to identify clusters of highly correlated genes, summarizing clusters, relating modules to sample traits, and for calculating module membership [47]. The genes adjacent to *TaIQDs* were found to be related to signaling pathways, cellular process, metabolic process, reproductive process, developmental process, and response to stimulus. To further identify the potential interactions of *TaIQD*s, we also constructed the protein–protein interaction (PPI) network of the corresponding *Arabidopsis* orthologs. It is noteworthy that *CIPK23* was found to be highly involved in WGCNA and PPI networks (Figure S8). In *Arabidopsis*, *CIPK23* functions in calcium (Ca^2+^)-related signaling pathways and is therefore involved in multiple physiological and developmental processes, such as iron acquisition [48], stomatal opening [49] and nutrient transporter [50]. Remarkably, two Ca^2+^ sensor proteins, namely CBL1 and CBL9, were identified as the upstream regulators of *CIPK23*. The CBL1/CBL9-CIPK23 complexes are required for activation of the K^+^ uptake channel *AKT1* and for enhanced K^+^ uptake under low K^+^ conditions [51]. These results suggested that *TaIQD*s might be involved in these signaling pathways, and additionally play essential roles in wheat growth, development, and various stress responses, but in-depth functional studies are needed.

### The asymmetric evolution patterns of *IQD* genes in hexaploid wheat

Plant polyploidization, together with the asymmetry in the process of co-evolution between different subgenomes, has contributed to sufficient genetic variation for environmental adaption [52]. A large number of studies have found that polyploid species have undergone asymmetric evolution in all aspects of their genomes. The draft genome of *Brassica oleracea* revealed the multi-layered asymmetrical evolution patterns between the *Brassica* subgenomes, such gene loss between subgenomes, amplification of tandem duplication and transposable elements, preferential enrichment for specific pathways and divergence in gene expression [52]. Asymmetric selection of defense-response genes also has led to ecotype change in *Brassica napus* [53]*.* More recent studies have provided evidence indicating that common wheat and cotton might have experienced asymmetric selection between different subgenomes [54, 55].

In order to find the asymmetric evolution patterns of *TaIQDs*, the member composition of the *IQD* gene families between common wheat and its progenitors was compared. For the A subgenome, a total of 4 (two for 2A and two for 7A) *IQD* genes were lost and 10 (three in 1A, two in 3A, two in 4A and three in 5A) were identified when compared with *Triticum urartu*. By contrast, only one in 1A and two in 3A IQDs were identified and one in 2A was lost after hexaploidization compared to *Triticum turgidum*. For the B subgenome, three *IQD* genes belonging to 2B, 3B and 4B were gained in hexaploid wheat. For the D subgenome, one more *IQD* gene on 1D was found in contrast to *Aegilops tauschii*. Noteworthy, the shorter the time taken by the ancestors to form the hexaploid wheat, the higher the consistency of the gene composition for *IQD* genes. In additions, previous studies have demonstrated that the gene number tend to be reversed towards diploid levels through gene loss following plant polyploidization [56]. In contrast, a slight increase was observed for *IQD* genes in wheat. We hypothesized that the exogenous introgression after the formation of hexaploid wheat may lead to the expansion of the TaIQD gene family, or any other complex mechanisms that remain unclear.

The presence of the homoeologous triads (composed of A, B and D genome copies) led us to examine the divergence from gene structure to biological function between subgenomes. The results indicated that around ~ 52.94% of the homoeologous genes from the A, B and D subgenomes have different predicted exon numbers. However*,* there was only 1 out of 34 homoeologous gene pairs *(TaIQD-1A/1B/1D-10)* that showed the divergent motif composition. Analysis of a total of 110 RNA-seq data revealed that most of the homoeologous genes showed a similar expression pattern. At the whole genome level, approximately 30% of the wheat homoeologous genes showed a biased expression pattern with lower or higher expression levels for a single homoeolog compared with the other two [31]. Regarding the *IQD* genes in wheat, a small portion of *TaIQDs* were differentially expressed in different stages/tissues or in response to exposure to stress. For example, *TaIQD-2A-5 and TaIQD-2D-5* were upregulated in salt stress*, TaIQD-2B-5* was downregulated in heat stress*.* Under cold treatment*, TaIQD-2A-5* was downregulated*,* and *TaIQD-2B-5*was upregulated*,* and *TaIQD-2D-5* was not expressed*.* These findings suggest the potential sub-functionalization or neo-functionalization of these genes.

As the most common type of genomic variation, SNPs have become an increasingly powerful molecular genetic marker for producing high-resolution genetic maps, linkage disequilibrium analysis, and marker-assisted breeding [57]. By taking advantage of the high-confidence *TaIQD*-related SNPs, the nucleotide diversity was calculated for each subgenomes with B > A > D in hexaploid wheat, which is basically consistent with previous studies [30, 58]*.* The asymmetric patterns of *IQD* genes in wheat will broaden our understanding on wheat genome evolution and will support research into the various important crops in the *Triticum* genus*.*

## Conclusions

This study comprehensively analyzed for the first time the wheat *IQD* genes. A total of 125 *TaIQDs* were thoroughly identified in the wheat genome. We also categorized the genes into four subgroups according to the phylogenetic relationships between wheat and maize, which was supported by the exon–intron structure and conserved motif composition analysis. The expression and co-expression analysis showed that the *TaIQDs* were widely involved in plant development, and in the response to environmental stresses. The expression of *TaIQD-2B-5*, *TaIQD-6B-2* and *TaIQD-6D-2* was significantly induced by exposure to various types of abiotic stresses, which might make these genes excellent targets for the molecular breeding of wheat. In addition, some of the *IQD* genes in the A, B and D subgenomes had different gene gain and loss rates, expression patterns and nucleotide diversity. Taken together, the findings of this study provide new insights into the biological function and molecular evolution of the *IQD* gene family in wheat during polyploidization.

## Materials and methods

### Identification of the *IQD* gene family in wheat

The sequence-related data of wheat were downloaded from the Ensembl Plants database (http://plants.ensembl.org/index.html). The previously reported IQD protein sequences from *Arabidopsis thaliana*, rice (*Oryza sativa*) and maize (*Zea mays*) were considered as reference sequences to blast against the proteins in the wheat whole genome using BLASTP with an e-value ≤ 1e-5 and identity ≥ 50%. In addition, the hidden Markov model (HMM) profiles of the IQ domain (Pfam ID: PF00612) were retrieved from the Pfam database (http://pfam.xfam.org/) and used as query to search the wheat proteins using HMMER v3.0 (https://www.ebi.ac.uk/Tools/hmmer/) with an e-value ≤ 0.01. The redundant sequences from BLAST and HMMER were manually removed and further verified using the online Pfam (http://pfam.xfam.org/search/sequence) databases. Only candidate proteins that had the IQ domain were retained. To confirm the presence of all candidate genes, a BLASTN search was conducted against the wheat expressed sequence tag (ESTs) downloaded from the National Center for Biotechnology Information (NCBI, Bethesda, MD, USA) database (https://www.ncbi.nlm.nih.gov/) using the following criteria: e-value ≤ 1e-5 and identity ≥ 80%. The physicochemical characteristics of TaIQD proteins, including molecular weight (MW), theoretical isoelectric point (pI) and grand average of hydropathy (GRAVY), were calculated by the online ExPASy server (https://web.expasy.org/protparam/). The subcellular localization of TaIQDs was predicted by the predictor tool in the Plant-mPLoc server (http://www.csbio.sjtu.edu.cn/bioinf/plant-multi/). The calmodulin target database (http://calcium.uhnres.utoronto.ca/ctdb/ctdb/sequence.html) was used to identify the putative CaM-binding sites of TaIQDs. *Arabidopsis* and rice orthologs were obtained using the program InParanoid v4.1.

### Phylogenetic analysis, gene structure and conserved motif analysis of TaIQD genes

The full-length IQD proteins from wheat as well as those from maize were used to generate multiple sequence alignment by ClustalX v1.83 with default parameters. The WebLogo tool (http://weblogo.berkeley.edu/logo.cgi) was used to display the sequence logo of the IQ motif. Then, the phylogenetic tree was constructed by the neighbor-joining (NJ) method using MEGAX v10.0 [59] with 1,000 replications, 95% partial deletion and a Poisson model. Ten motifs were scanned using MEME v.5.0.5 with a width ranging from 8 to 50 amino acids [60] (http://meme-suite.org/tools/meme). The Gene Structure Display Server [61] (http://gsds.cbi.pku.edu.cn/) was used to visualize the exon–intron composition of *TaIQD* genes.

### Gene duplication, homoeologous relationships and ka/ks estimation

The chromosomal location of *TaIQDs* was obtained from the wheat genome annotation file (http://plants.ensembl.org/index.html) and diagrams were drawn using MG2C v2.1 (http://mg2c.iask.in/mg2c_v2.0/). In order to establish the syntenic relationships of *IQD* genes among the A, B and D subgenomes in wheat, we performed an all *vs*. all BLASTP search for all the TaIQD proteins. TaIQDs clustered within the same branches of the phylogenetic tree and displaying more than 95% similarity between each other were considered as homoeologous groups. According to their chromosome location, MCScanX was used to determine the syntenic relationships of the *TaIQD* gene family among the A, B and D subgenomes [62].

To evaluate the evolutionary relationships of *IQD* genes between wheat and its relatives (*Triticum urartu*, *Triticum dicoccoides*, *Triticum turgidum* and *Aegilops tauschii*), the proteins of these four species were retrieved from the Ensembl Plants database. Then, we used the same methods and criteria as described for wheat to identify the *IQD* genes in *Triticum urartu*, *Triticum dicoccoides*, *Triticum turgidum* and *Aegilops tauschii*. The synteny analysis of *IQD* genes between the homologs of wheat and its relatives was performed using MCScanX. The syntenic maps were visualized using Circos v0.67. The Ka (non-synonymous substitution)/Ks (synonymous substitution) ratio value was calculated using the PAML v4.9e package to estimate the divergence of the homologous genes [63].

### Analysis of the expression profiles of *TaIQD* genes using transcriptome data

A total of 110 spatiotemporal and stress treatment RNA-seq samples of wheat, including root, stem, leaf, young spike (PRJNA525250), embryonal stage (PRJNA532455), 3 to 26 days after anthesis (DAA) leaves (PRJNA497810), and abiotic stresses (cold, heat, drought, salt, and P and iron starvation) (PRJNA253535, PRJNA257938, PRJNA487922 and PRJNA529036), were downloaded from the NCBI Sequence Read Archive (SRA) database. Detailed sample information is listed in Table S13. The fragments per kilobase per million (FPKM) value was calculated using HISAT2 v2.1.0 and the StringTie v1.3.5 pipeline. The log_2_ (FPKM + 1) value of *TaIQDs* was used to generate the expression heatmaps using the pheatmap package in R. We used τ (Tau) as a measure of the tissue specificity [64]. The τ values ranged from 0 to 1, with a large value representing high tissue specificity and a low value representing low tissue specificity. We considered τ > 0.6 as high tissue-specificity.

### *Cis*-acting elements and regulatory network analysis

The 1.5 kb upstream DNA sequences from the gene transcription initiation site of *TaIQDs* were extracted and submitted to the PlantCARE online database (http://bioinformatics.psb.ugent.be/webtools/plantcare/html/) to search for the putative *cis*-acting elements within the promoter region. The cDNA sequences of *TaIQD* genes were uploaded to the psRNATarget (http://plantgrn.noble.org/psRNATarget/) to find the candidate miRNA target sites. The co-expression network between *TaIQD*s and other related genes were constructed using the weighted gene co-expression network analysis (WGCNA) package in R according to their expression levels generated by the RNA-seq data. The co-expression relationships with weighted values larger than 0.25 were retained for subsequent analysis. The PPI network was also constructed based on the orthologous of *TaIQD*s in *Arabidopsis* using the STRING online tools (https://string-db.org/). The Gene Ontology (GO) and Kyoto Encyclopedia of Genes and Genomes (KEGG) enrichment analyses of the co-expressed genes were performed using the KOBAS software (http://bioinfo.org/kobas), the potential relevancy between *TaIQDs* and other wheat genes was visualized using Cytoscape v3.8.0.

### Plant material, stress treatment, RNA extraction and qRT-PCR analysis

Seeds of *Triticum aestivum* landrace ‘Chinese Spring’ were geminated on petri dishes under dark condition, and cultured in the growth chamber with a 16 h light/8 h dark cycle at 23 ± 1 °C. The three-leaf stage seedlings were subjected to abiotic treatments. For salt, drought, cold and heat stresses, the plants were subjected to treatment with 150 mM NaCl, 20% PEG-6000, at 4℃ and 42℃, respectively. The leaves were sampled at 0, 1, 6, 12 and 24 h with three independent biological replications, and the untreated plants were used as controls. The sampled leaves were rapidly pre-cooled in liquid nitrogen and then stored at -80℃ for subsequent analysis.

The plant RNA Kit Reagent (Omega Bio-Tek Inc., Norcross, GA, USA) was used to isolate total RNA. The cDNA was synthesized using the Evo M-MLV RT Mix Kit (Accurate Biology, Changsha, China). The quantitative real-time polymerase chain reaction (qRT-PCR) analysis was performed using a SYBR® Green Premix Pro Taq HS qPCR Kit (Accurate Biology) on an Applied Biosystems™ 7500 Real-Time PCR System (Thermo Fisher Scientific Inc., Waltham, MA, USA). The Primer Premier v5.0 software was used to design the primer sequences for *TaIQD* genes. *The Elongation Factor 1-Alpha* gene was used as the internal control. The primer information is listed in Table S14. The PCR cycling conditions were as follows: 95℃ for 30 s for 1 cycle, followed by 40 cycles of 95℃ for 5 s, 60℃ for 30 s. The 2^−ΔΔCT^ method was used to evaluate the mRNA relative expression levels of *TaIQDs* [65]. The significance analysis was performed using the t-test package in R. The 0.05 and 0.01 significance level are indicated by one asterisk (*) and two asterisks (**), respectively.

### Nucleotide variation and population structure analysis

The early released whole-genome sequencing population from the genera *Triticum* and *Aegilops* were used to identify the nucleotide variation of *TaIQD* genes. The genomic variation data was downloaded from the Genome Variation Map database under accession number GVM000082 [30]. The following criteria were used for filtration: minor allele frequency (MAF) > 0.05, and maximum missing rate < 0.1. To avoid the difference due to sampling, the same number of samples for different population was selected as much as possible. Therefore, we use a total of 27 *Triticum rartu*, 28 *Triticum dicoccoides*, 26 *Triticum dicoccum*, 10 *Triticum turgidum*, 26 *Triticum aestivum* (landrace), 20 *Triticum aestivum* (cultivar) and 30 *Aegilops tauschii* (Table S15). The PCA was performed using the smartpca subroutine in EIGENSOFT v6.1.4 [66]. An unrooted phylogenetic tree was generated by the neighbor-joining method with the parameters (1,000 bootstrap replications, 95% partial deletion and Poisson model), using the MEGAX v10.0 software. The population structure was analyzed by Admixture v1.3 [67] with the following parameters: the number of subgroups K ranged from 2 to 10, 10,000 times iteration and each K value was repeated five times. The VCFtools v0.1.17 genome toolbox was used to calculate the nucleotide diversity (π) and fixation index (*F*_*st*_). The above-mentioned analysis was performed for the A, B and D subgenomes, respectively.

## Supplementary Information

**Additional file 1: Figure S1.** The predicted ten motifs of TaIQD proteins based on MEME online software.

**Additional file 2: Figure S2.** The chromosomal location of TaIQDs in the wheat genome.

**Additional file 3: Figure S3.** Syntenic relationships of IQD genes among A, B and D subgenomes in common wheat.

**Additional file 4: Figure S4.** qRT-PCR analysis of TaIQDs in response to salt, drought, heat and cold treatments. Error bars indicate standard errors from three independent replications. One asterisk (*) indicates 0.05 significance level. Two asterisks (**) indicates 0.01 significance level.

**Additional file 5: Figure S5.** Analysis of the representative cis-regulatory elements in the promoter regions of TaIQDs.

**Additional file 6: Figure S6.** Phylogenetic relationships, PCA and population structure analysis for the group B genomes based on TaIQD-related SNPs. The SNPs from the B subgenome of Triticum dicoccoides, Triticum dicoccum, Triticum turgidum and Triticum aestivum were used. A: Neighbor-joining phylogenetic tree, B: PCA analysis of PC1 vs PC2, C: PCA analysis of PC1 vs PC3, D: Population structure was estimated by ADMIXTURE with the K range from 2 to 8.

**Additional file 7: Figure S7.** Phylogenetic relationships, PCA and population structure analysis for the group D genomes based on TaIQD-related SNPs. The SNPs from the D subgenome/genome of Triticum dicoccoides, Triticum dicoccum, Triticum turgidum and Triticum aestivum were used. A: Neighbor-joining phylogenetic tree, B: PCA analysis of PC1 vs PC2, C: PCA analysis of PC1 vs PC3, D: Population structure was estimated by ADMIXTURE with the K range from 2 to 8.

**Additional file 8: Figure S8.** The protein-protein interaction (PPI) network of TaIQDs according to the orthologs in Arabidopsis.

**Additional file 9: Supplementary tables. Table S1.** Validation of the IQ domain using the SMART, HMMER, and NCBI-CDD online databases. **Table S2.** Characteristics of IQD gene family in common wheat. **Table S3.** Conserved amino acids composition of IQ domain in different species. **Table S4.** Predicted calmodulin-binding sites in wheat IQD proteins. **Table S5.** Distribution of subgroup members of IQDs in different species. **Table S6.** The Ka/Ks ratios for syntenic TaIQD genes. **Table S7.** Characteristics of IQD gene family in wheat progenitors. **Table S8.** The Ka/Ks ratios for syntenic IQD genes in common wheat and its progenitors. **Table S9.** Characteristics of cis-acting regulatory elements in the promoter regions of TaIQD genes. **Table S10.** List of putative miRNAs that targeted to TaIQD genes. **Table S11.** Genomic distribution of nucleotide variations of TaIQD genes. **Table S12.** TW test of TaIQD-related genes for A, B and D subgenomes. **Table S13.** Accession numbers and samples information of RNA-seq data used in this study. **Table S14.** The primers information used in the qRT-PCR analysis. **Table S15.** Passport information of the resequencing accessions used in this study.

## Data Availability

All data supporting the conclusions of this article are provided within the article and its additional files. The sequences of *Arabidopsis thaliana*, *Oryza sativa*, *Triticum urartu*, *Triticum dicoccoides*, *Triticum turgidum*, *Aegilops tauschii* and *Triticum aestivum* are available in the Ensemble Plants database (http://plants.ensembl.org/index.html). The gene expression data was downloaded from the NCBI database (http://www.ncbi.nlm.nih.gov/geo/) under accession number PRJNA525250, PRJNA497810, PRJNA532455, PRJNA257938, PRJNA529036, PRJNA253535 and PRJNA487922. The genomic variation data was downloaded from the SnpHub (http://wheat.cau.edu.cn/WheatUnion/b_4/).

## Abbreviations

aa: Amino acids
ABA: Abscisic acid
CaM: Calmodulin
CML: CaM-like
CBL: Calcineurin B-like
CDPK: Ca^2^^+^-dependent protein kinase
CaMBP: Calmodulin-binding protein
CaMBD: Calmodulin-binding domain
DAA: Days after anthesis
EST: Expressed sequence tag
FPKM: Fragments per kilobase per million
*F*_*st*_: Fixation index
GA: Gibberellin
GO: Gene Ontology
GRAVY: Grand average of hydropathy
HMM: Hidden Markov Model
IQD: IQ67-domain
Ka: Non-synonymous substitution
Ks: Kynonymous substitution
MAF: Minor allele frequency
MeJA: Methyl jasmonate
MW: Molecular weight
NJ: Neighbor-joining
PCA: Principal components analysis
pI: Theoretical isoelectric point
PPI: Protein-protein interaction
qRT-PCR: Quantitative real time polymerase chain reaction
SA: Salicylic acid
SNP: Single nucleotide polymorphism
SRA: Sequence Read Archive
WRE3: Wounding responsive element 3
π: Nucleotide diversity
