# Supplementary material for: Genome-wide identification, expression analysis and evolutionary relationships of the IQ67-domain gene family in common wheat (Triticum aestivum L.) and its progenitors
Source: BMC Genomics. 2022 Apr 5;23:264. doi: 10.1186/s12864-022-08520-w (PMC8981769; doi:10.1186/s12864-022-08520-w)
Supplement: Supplementary file 1 — Additional file 1: Figure S1. The predicted ten motifs of TaIQD proteins based on MEME online software. [file 12864_2022_8520_MOESM1_ESM.pdf]

Logo

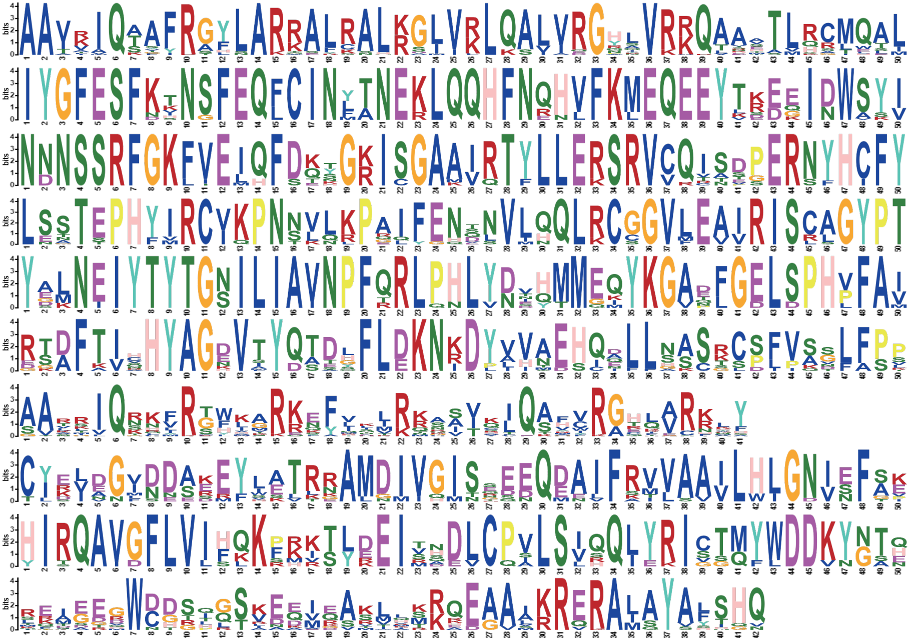

Motif	E-value	Sites	Width
Motif 1	2.4e-2599	75	50
Motif 2	4.9e-1286	28	50
Motif 3	3.9e-1124	27	50
Motif 4	1.3e-1122	28	50
Motif 5	5.4e-911	22	50
Motif 6	5.4e-907	28	50
Motif 7	1.7e-818	46	41
Motif 8	4.8e-863	28	50
Motif 9	2.3e-794	22	50
Motif 10	5.5e-767	38	42
