# Supplementary figures and images for: Genome-wide identification, expression analysis and evolutionary relationships of the IQ67-domain gene family in common wheat (Triticum aestivum L.) and its progenitors
Source: BMC Genomics. 2022 Apr 5;23:264. doi: 10.1186/s12864-022-08520-w (PMC8981769; doi:10.1186/s12864-022-08520-w)

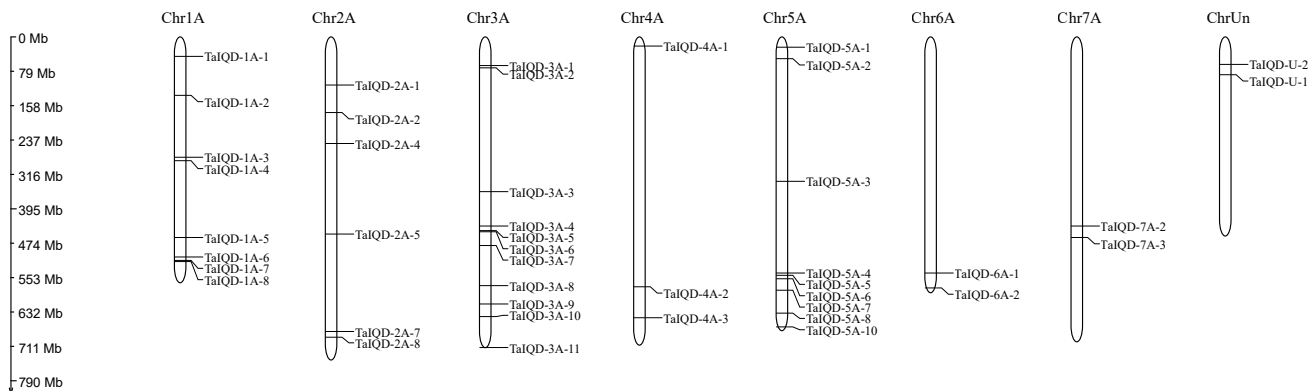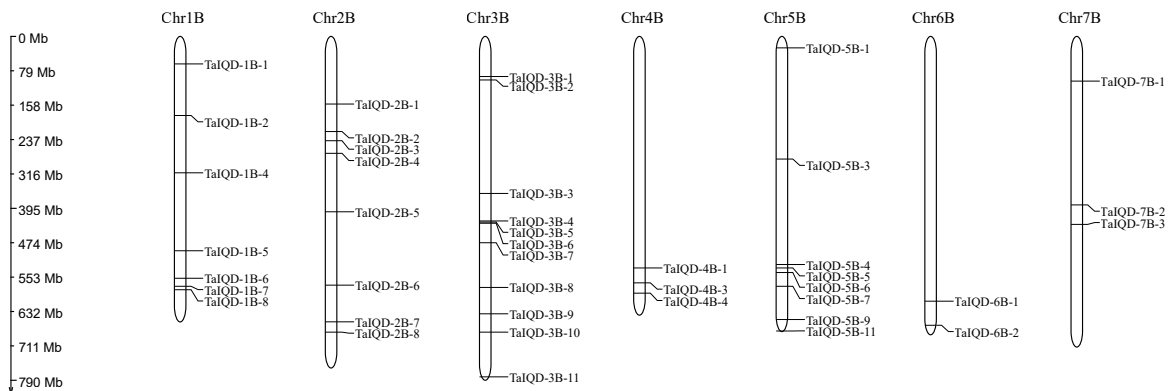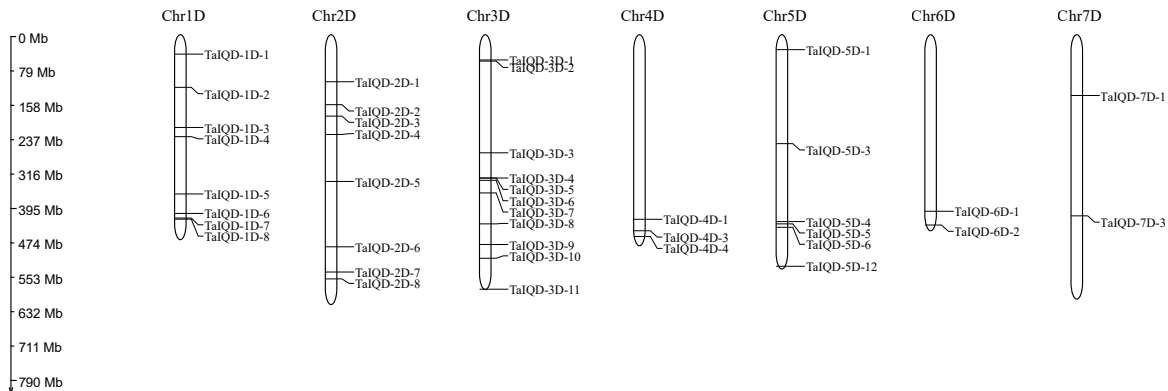

Supplement: Supplementary file 2 — Additional file 2: Figure S2. The chromosomal location of TaIQDs in the wheat genome. [file 12864_2022_8520_MOESM2_ESM.pdf]

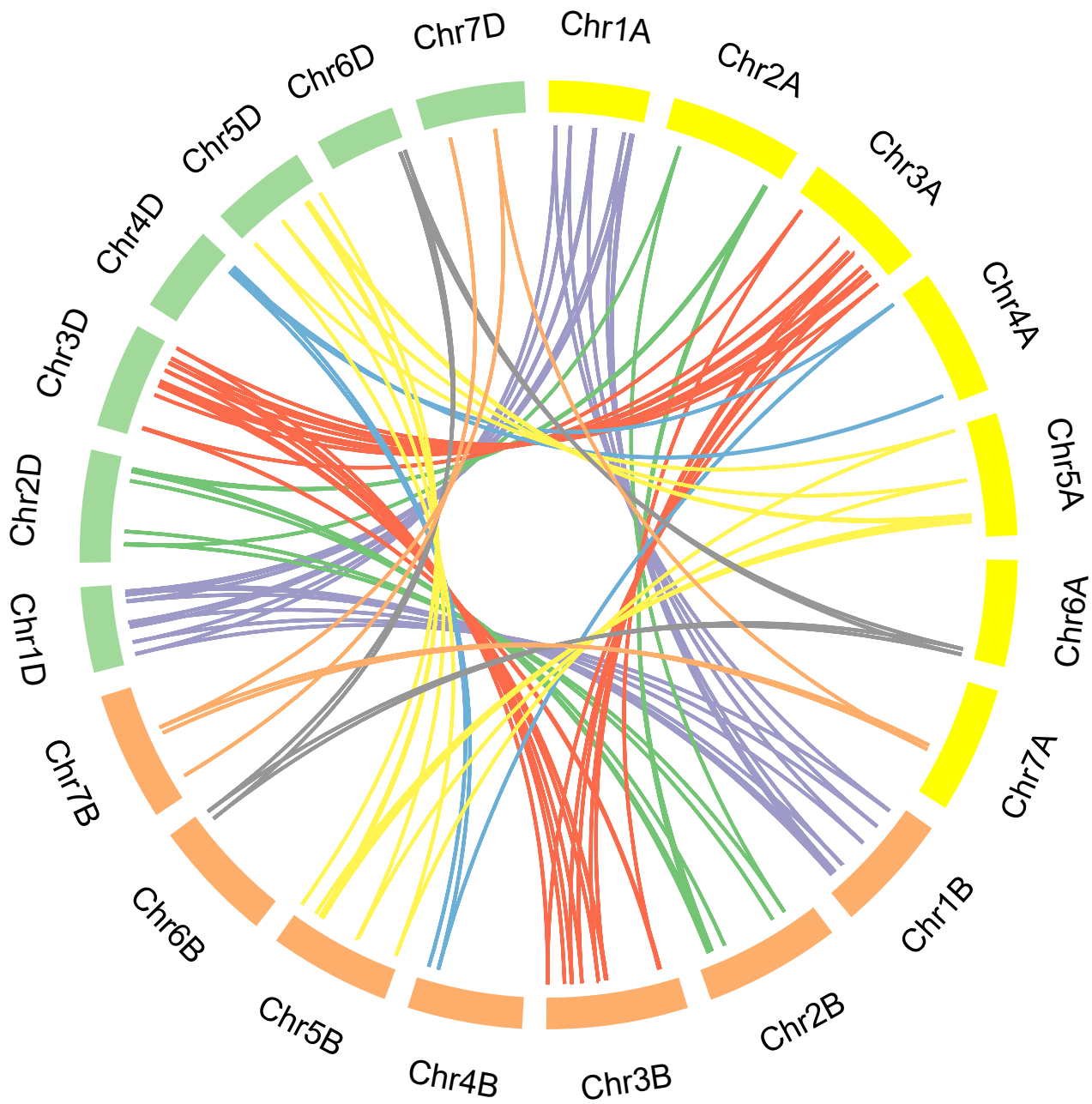

Supplement: Supplementary file 3 — Additional file 3: Figure S3. Syntenic relationships of IQD genes among A, B and D subgenomes in common wheat. [file 12864_2022_8520_MOESM3_ESM.pdf]

TaIQD-1A-7

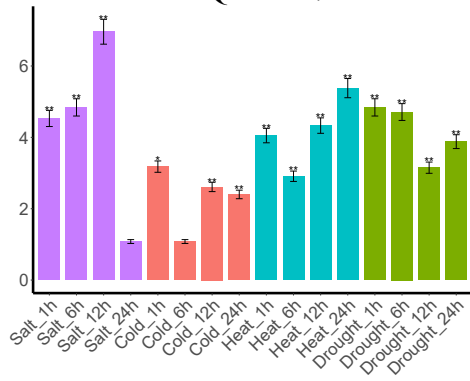

TaIQD-2A-2

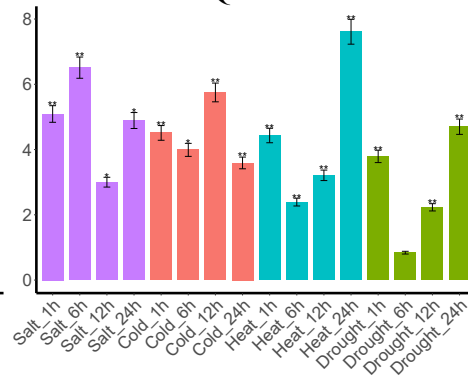

TaIQD-3A-9

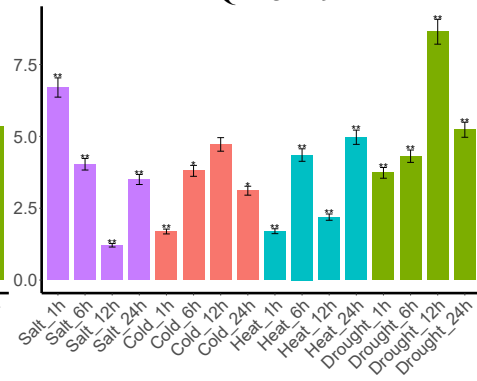

TaIQD-3B-11

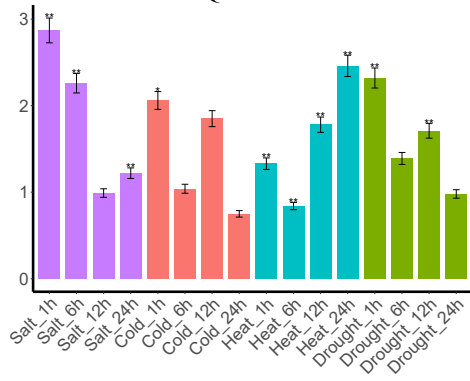

TaIQD-3B-5

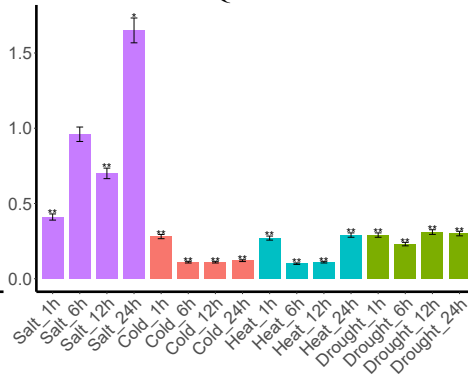

TaIQD-3D-10

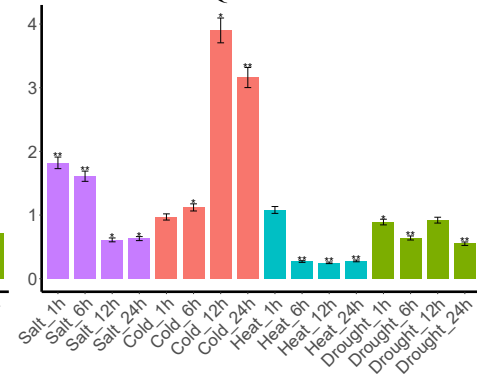

TaIQD-3D-9

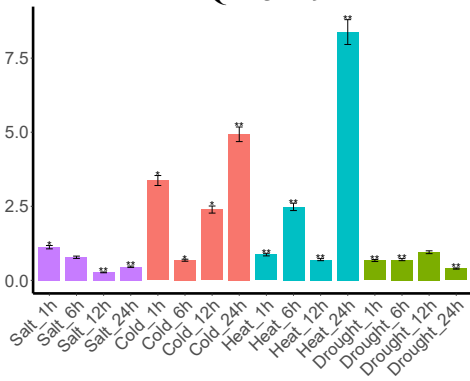

TaIQD-5A-6

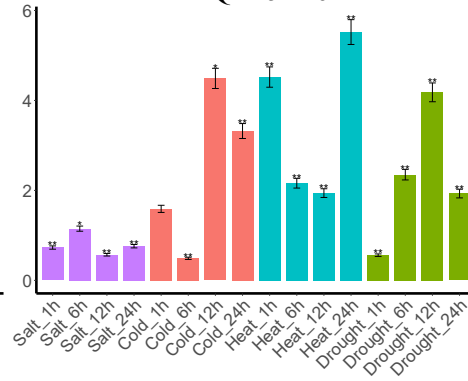

TaIQD-5B-9

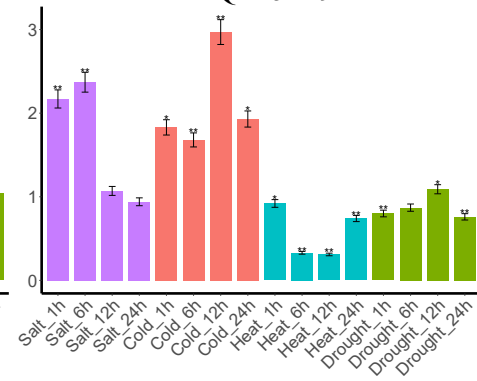

■ Cold  
■ Drought  
■ Heat  
■ Salt

Supplement: Supplementary file 4 — Additional file 4: Figure S4. qRT-PCR analysis of TaIQDs in response to salt, drought, heat and cold treatments. Error bars indicate standard errors from three independent replications. One asterisk (*) indicates 0.05 significance level. Two asterisks (**) indicates 0.01 significance level. [file 12864_2022_8520_MOESM4_ESM.pdf]

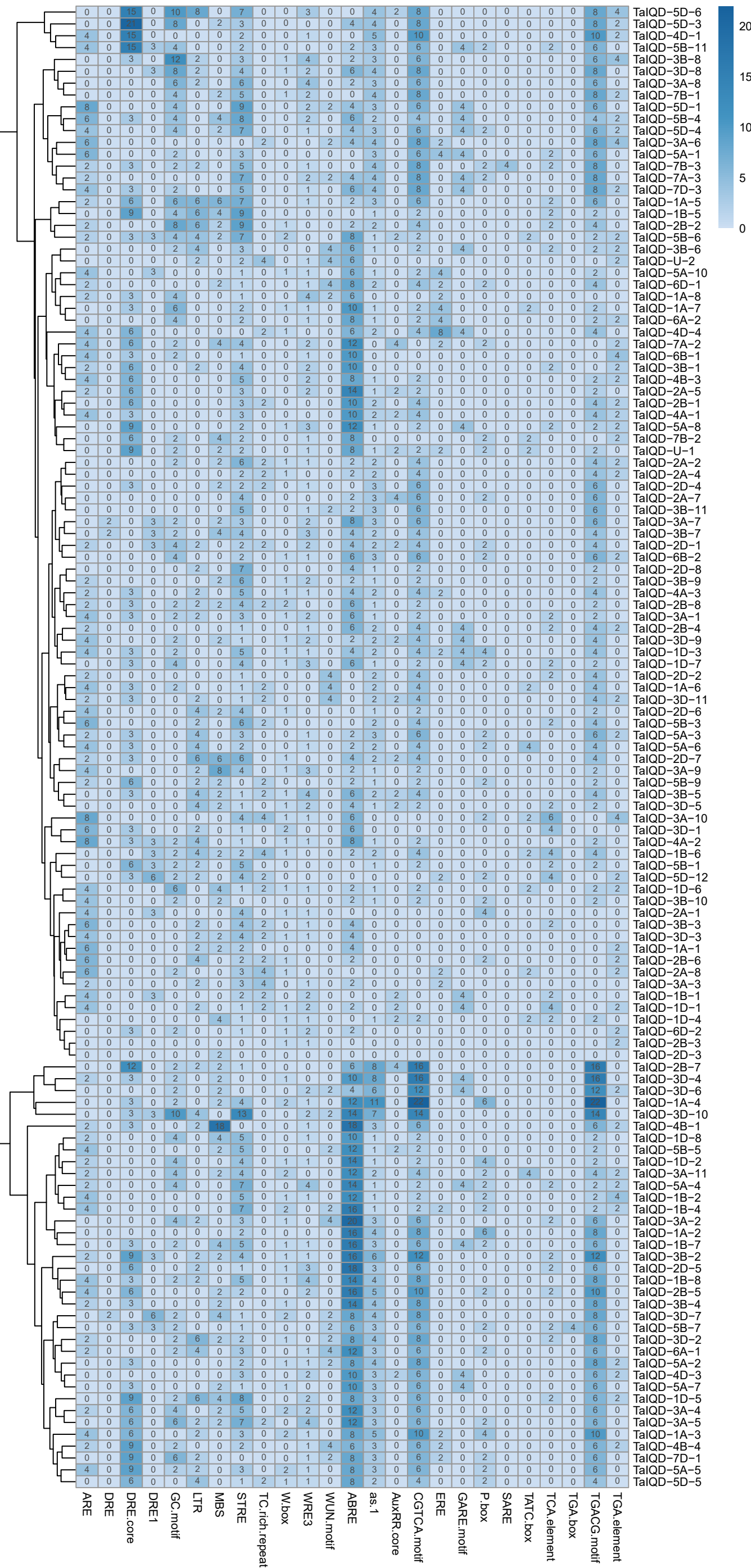

Supplement: Supplementary file 5 — Additional file 5: Figure S5. Analysis of the representative cis-regulatory elements in the promoter regions of TaIQDs. [file 12864_2022_8520_MOESM5_ESM.pdf]

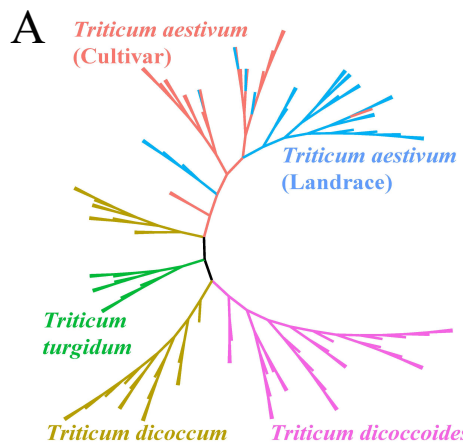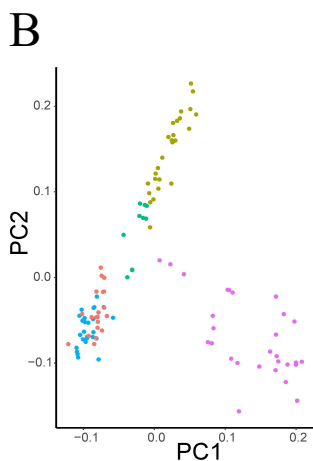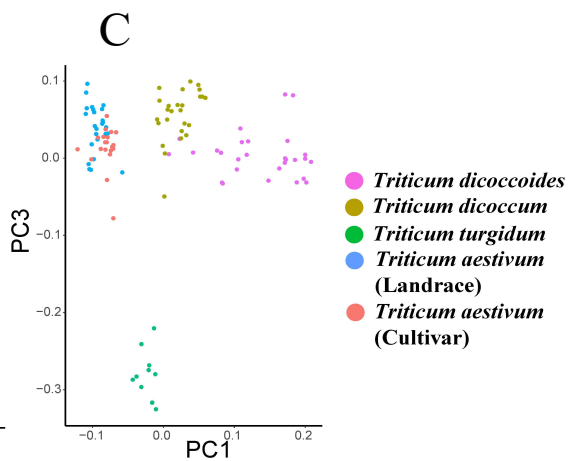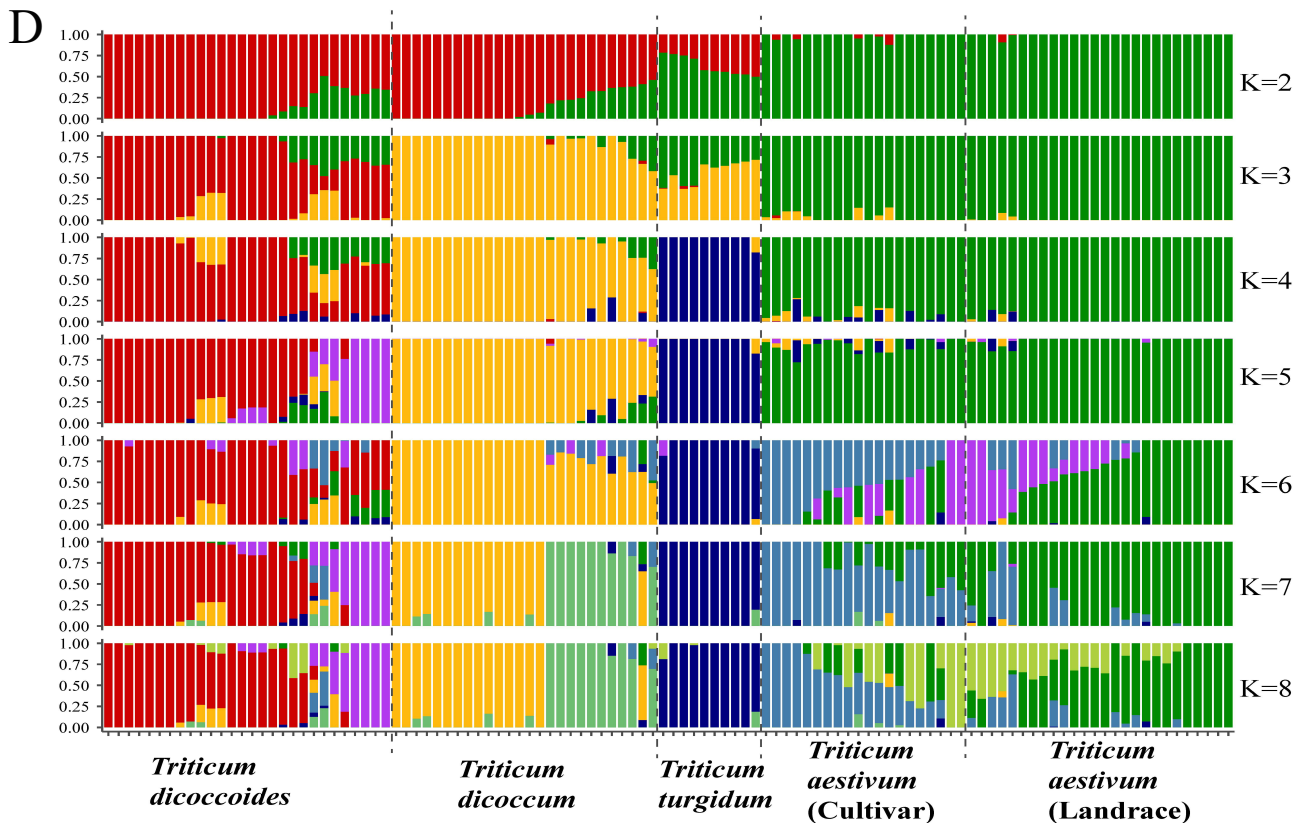

Supplement: Supplementary file 6 — Additional file 6: Figure S6. Phylogenetic relationships, PCA and population structure analysis for the group B genomes based on TaIQD-related SNPs. The SNPs from the B subgenome of Triticum dicoccoides, Triticum dicoccum, Triticum turgidum and Triticum aestivum were used. A: Neighbor-joining phylogenetic tree, B: PCA analysis of PC1 vs PC2, C: PCA analysis of PC1 vs PC3, D: Population structure was estimated by ADMIXTURE with the K range from 2 to 8. [file 12864_2022_8520_MOESM6_ESM.pdf]

A

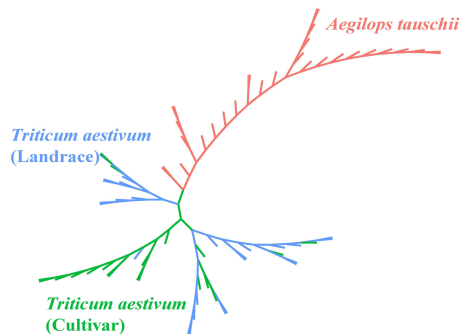

B

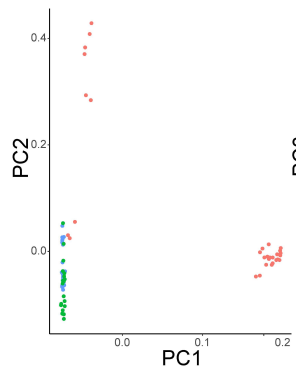

C

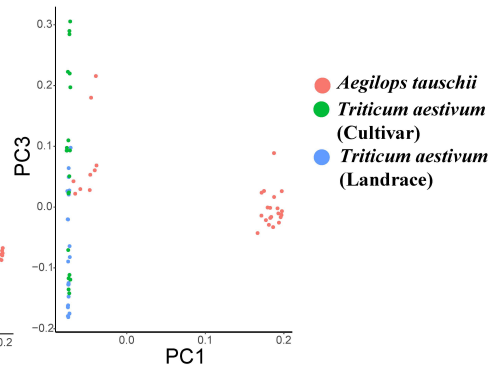

D

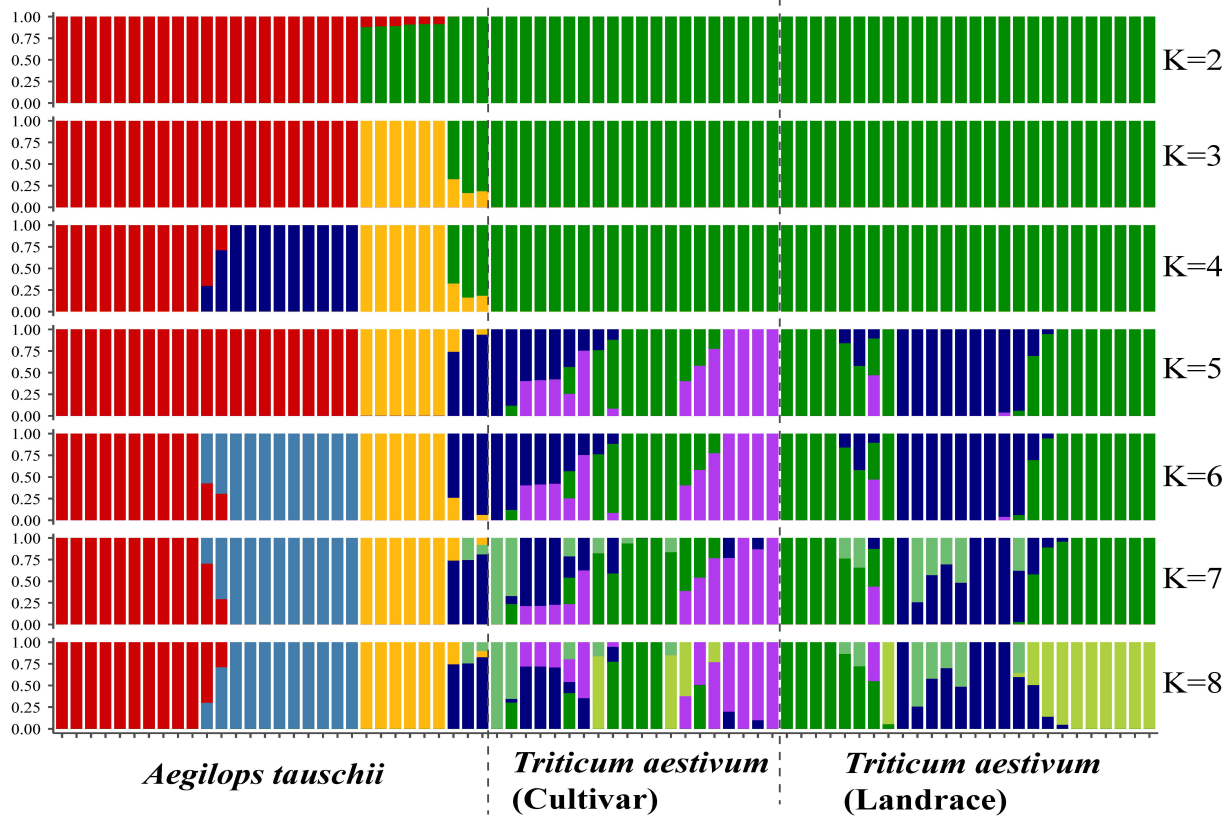

Supplement: Supplementary file 7 — Additional file 7: Figure S7. Phylogenetic relationships, PCA and population structure analysis for the group D genomes based on TaIQD-related SNPs. The SNPs from the D subgenome/genome of Triticum dicoccoides, Triticum dicoccum, Triticum turgidum and Triticum aestivum were used. A: Neighbor-joining phylogenetic tree, B: PCA analysis of PC1 vs PC2, C: PCA analysis of PC1 vs PC3, D: Population structure was estimated by ADMIXTURE with the K range from 2 to 8. [file 12864_2022_8520_MOESM7_ESM.pdf]

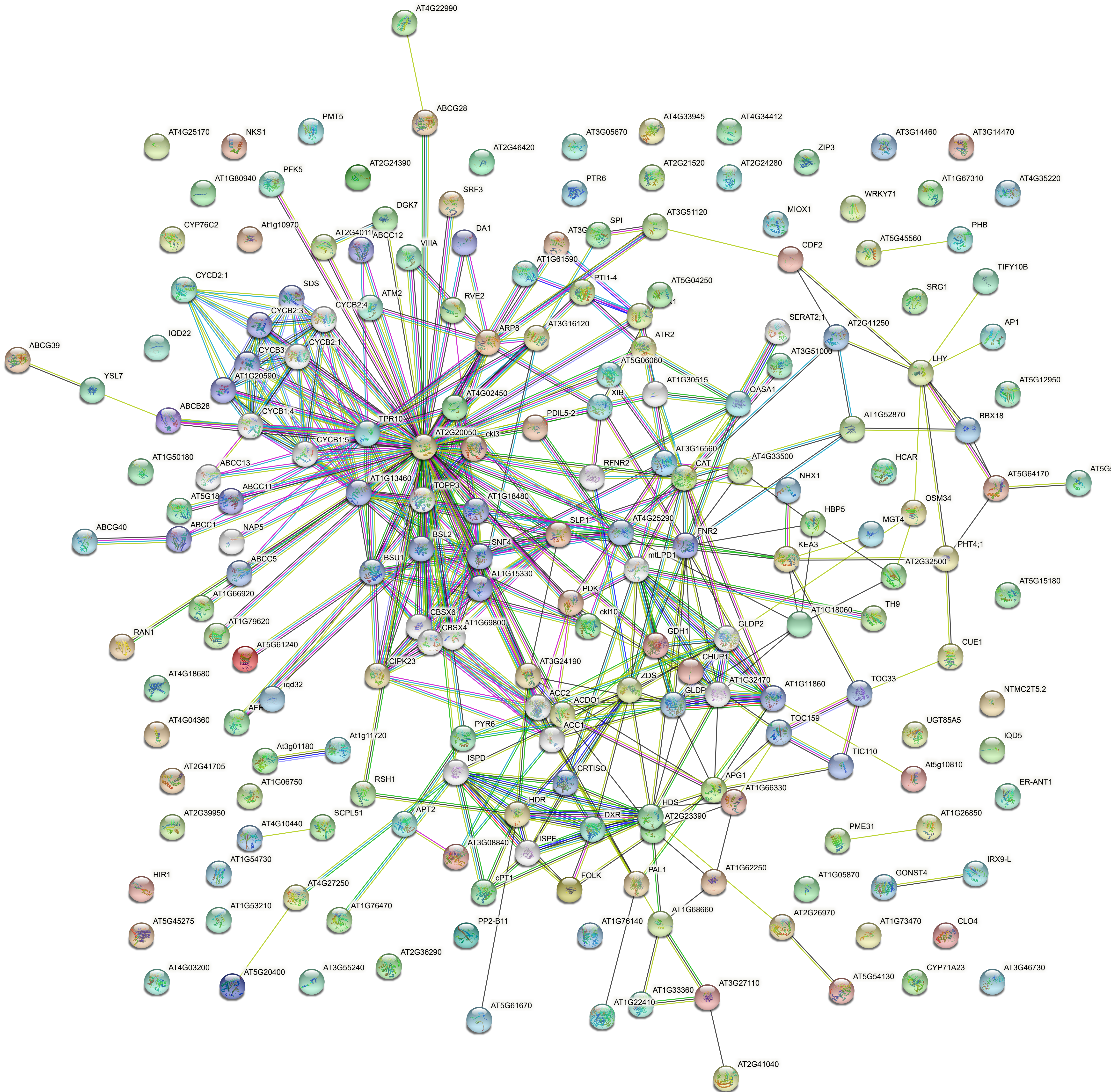

Supplement: Supplementary file 8 — Additional file 8: Figure S8. The protein-protein interaction (PPI) network of TaIQDs according to the orthologs in Arabidopsis. [file 12864_2022_8520_MOESM8_ESM.pdf]
